# Protein structure determination by single-wavelength anomalous diffraction phasing of X-ray free-electron laser data

**DOI:** 10.1107/S2052252516002980

**Published:** 2016-03-09

**Authors:** Karol Nass, Anton Meinhart, Thomas R. M. Barends, Lutz Foucar, Alexander Gorel, Andrew Aquila, Sabine Botha, R. Bruce Doak, Jason Koglin, Mengning Liang, Robert L. Shoeman, Garth Williams, Sebastien Boutet, Ilme Schlichting

**Affiliations:** a Department of Biomolecular Mechanisms, Max Planck Institute for Medical Research, Jahnstrasse 29, 69120 Heidelberg, Germany; b European XFEL GmbH, Albert-Einstein-Ring 19, 22761 Hamburg, Germany; c SLAC National Accelerator Laboratory, 2575 Sand Hill Road, Menlo Park, CA 94025, USA

**Keywords:** serial femtosecond crystallography, SFX, X-ray free-electron lasers, XFELs, SAD phasing, single-wavelength anomalous diffraction

## Abstract

Serial femtosecond crystallography (SFX) at X-ray free-electron lasers (XFELs) offers unprecedented possibilities for macromolecular structure determination of systems that are prone to radiation damage. However, phasing XFEL data *de novo* is complicated by the inherent inaccuracy of SFX data, and only a few successful examples, mostly based on exceedingly strong anomalous or isomorphous difference signals, have been reported. Here, it is shown that SFX data from thaumatin microcrystals can be successfully phased using only the weak anomalous scattering from the endogenous S atoms. Moreover, a step-by-step investigation is presented of the particular problems of SAD phasing of SFX data, analysing data from a derivative with a strong anomalous signal as well as the weak signal from endogenous S atoms.

## Abstract 2

A systematic analysis of anomalous diffraction data obtained by serial femtosecond crystallography at an X-ray free-electron laser is presented and sulfur SAD phasing of SFX data from thaumatin microcrystals is demonstrated.

## 1. Introduction   

X-ray crystallography is a highly successful method for the determination of three-dimensional structures of molecules. In slightly more than a hundred years since its establishment, crystallography has undergone a great deal of development, extending the size of the molecules investigated from small inorganic compounds to megadalton protein machines. This progress has proceeded hand in hand with the development of instrumentation and X-ray sources, the latest of which are X-ray free-electron lasers (XFELs). XFELs are linear accelerator-based large-scale facilities that provide femto­second X-ray pulses with a peak brilliance that exceeds that of third-generation synchrotron sources by nine orders of magnitude. These short, intense pulses allow diffraction data to be collected before the onset of significant radiation damage (‘diffraction before destruction’; Neutze *et al.*, 2000 ▸; Chapman *et al.*, 2011 ▸; Boutet *et al.*, 2012 ▸), notably for systems that are prone to damage, such as metalloproteins or microcrystals in general. Examples to date include the structure determination of microcrystals grown *in vivo* (Redecke *et al.*, 2013 ▸; Sawaya *et al.*, 2014 ▸) and in lipidic cubic phase (Kang *et al.*, 2015 ▸; Liu *et al.*, 2013 ▸; Zhang *et al.*, 2015 ▸) and of metalloproteins such as photosystem II (Suga *et al.*, 2015 ▸), cytochrome *c* oxidase (Hirata *et al.*, 2014 ▸) and peroxidase (Chreifi *et al.*, 2016 ▸). However, all of these successes relied on prior phase information, whereas progress with *de novo* phasing of XFEL diffraction data has been sparse (Barends *et al.*, 2014 ▸; Yamashita *et al.*, 2015 ▸; Nakane *et al.*, 2015 ▸). This is owing to the nature of the data collected with XFEL sources and the lack of accuracy of the integrated intensities provided by current analysis programs. XFEL data collection differs from conventional rotation or oscillation data acquisition in several respects. The femtosecond exposure time precludes any rotation during exposure and thus results in the collection of still images that contain only partial reflections. Since exposure to the unattenuated XFEL beam destroys all or part of the crystal being illuminated, a new crystal, or a fresh part thereof, is required for the next exposure. In the case of microcrystals, this must necessarily be a fresh, randomly oriented crystal, leading to a data-collection approach termed serial femtosecond crystallography (SFX). Since microcrystal size and quality often vary and since crystals can be exposed anywhere between the periphery and the centre of the X-ray beam focal spot, the diffracted intensities can vary from shot to shot even if the microcrystals are oriented identically. Moreover, both the pulse energy (intensity) and the photon-energy (wavelength) distributions of the FEL pulses vary from shot to shot. All of this results in significant fluctuations in the intensity measurements.

A number of programs for SFX data analysis are available to date (Kabsch, 2014 ▸; Sauter *et al.*, 2013 ▸; Uervirojnangkoorn *et al.*, 2015 ▸; White *et al.*, 2012 ▸, Ginn, Brewster *et al.*, 2015 ▸; Ginn, Messerschmidt *et al.*, 2015 ▸). It has been shown that Monte Carlo integration can be used to average out the various fluctuations by using a large number of measurements (Kirian *et al.*, 2010 ▸). Similarly, partial intensities also converge to their real values when the multiplicity is high. It has been demonstrated that these intensities are accurate enough for *de novo* structure determination. In a pioneering proof-of-principle study, Barends *et al.* (2014 ▸) utilized the anomalous signal from microcrystals of a gadolinium derivative of lysozyme collected at the Linac Coherent Light Source (LCLS) to phase the structure by the single-wavelength anomalous diffraction (SAD) method. Successful phasing, followed by fully automated model building, required 60 000 indexed diffraction patterns. Yamashita *et al.* (2015 ▸) described the *de novo* structure determination of luciferin-regenerating enzyme (LRE) using data sets from microcrystals of the native protein and of a highly isomorphous mercury derivative, both collected at the SPring-8 Angstrom Compact Free-Electron Laser (SACLA). They phased their structure using the method of single isomorphous replacement with anomalous scattering (SIRAS). In this case, a total of 20 000 indexed patterns, 10 000 each for the native and the derivative, sufficed for phasing provided that 1.7 Å resolution data were used. In both of these *de novo* phasing approaches the intensity differences employed for phasing were of the order of 10%. In the lysozyme SAD case this was owing to the extremely strong anomalous scattering from two gadolinium ions above the *L*_III_ edge (*f*′′ = 12.5 e at 8.5 keV). In the case of the luciferin-regenerating enzyme SIRAS study it was primarily the isomorphous differences between the native and mercury-derivative data that were utilized, although the anomalous signal above the *L*_III_ edge of the strong anomalous scattering from the bound Hg atom (*f*′′ = 9.7 e at 12.6 keV) was also exploited. While both cases represent important methodological steps towards routine *de novo* phasing of SFX data, neither is typical. The anomalous signal of the Gd derivative of lysozyme is exceptionally strong and the high isomorphism of the native and Hg-derivative SFX data sets of the luciferin-regenerating enzyme microcrystals (the difference between the unit-cell parameters was reported to be smaller than 0.2%; Yamashita *et al.*, 2015 ▸), combined with very high resolution diffraction, is rarely observed for challenging structure determinations. Most importantly, both approaches relied on the successful derivatization of the native crystals, whereas in reality finding a suitable derivative is often difficult.

The use of sulfur as an endogenous source of anomalous scattering circumvents all of the problems associated with the search for and use of heavy-atom derivatives (see, for example, Hendrickson & Teeter, 1981 ▸; Dauter *et al.*, 1999 ▸; Liu *et al.*, 2012 ▸, 2014 ▸; Akey *et al.*, 2014 ▸; Liu & Hendrickson, 2015 ▸; Weinert *et al.*, 2015 ▸; Rose *et al.*, 2015 ▸). The disadvantage of native phasing is that typical sulfur or phosphorus anomalous signals are of the order of only 1–2%, requiring much higher data accuracy than for heavy-atom derivatives. Consequently, it was unclear whether phasing of XFEL data by sulfur SAD would be feasible. To test this, we carried out SFX measurements using thaumatin as a well established model system (Watanabe *et al.*, 2005 ▸; Mueller-Dieckmann *et al.*, 2005 ▸). Recently, Nakane *et al.* (2015 ▸) published a seminal demonstration of the native sulfur phasing of lysozyme from SFX data collected at SACLA. As expected for XFEL data, a large number of images (150 000) was required to obtain sufficiently accurate data for sulfur SAD phasing, but only a limited analysis of the data was provided.

Here, we describe the particular challenges of native sulfur phasing of SFX data collected at XFELs in much greater detail. However, before embarking on this analysis, we first revisited the lysozyme Gd-derivative data and exploited their large anomalous signal and explored whether the elimination of systematic errors could reduce the number of indexed patterns that are required for SAD phasing. Indeed, by optimizing the geometry (metrology) of the multi-tiled detector, by accounting for small changes in the sample-to-detector distance and by scaling the data, we could successfully phase the lysozyme Gd-derivative data using only ∼7000 indexed patterns as opposed to the previously required 60 000 (Barends *et al.*, 2014 ▸). Applying the same meticulous methodology to our thaumatin data, we were able to phase SFX data using the small anomalous signal of sulfur and automatically build an almost complete molecular model using 125 000 indexed patterns of thaumatin microcrystals. We expect that this number will decrease significantly with further improvements in SFX analysis software, in particular to account for the partiality of the reflections, to apply specially adapted implementations of profile fitting, for example as demonstrated by Kabsch (2014 ▸), and to make use of the spectral information of the FEL pulse.

## 2. Methods   

### 2.1. Crystallization of thaumatin and the lysozyme Gd derivative   

*Thaumatococcus daniellii* thaumatin was purchased from Sigma–Aldrich Chemie GmbH (Schnelldorf, Germany). Macroscopic crystals were obtained at room temperature by vapour diffusion in Linbro plates, equilibrating hanging drops consisting of equal volumes of protein solution (40 mg ml^−1^ in 0.1 *M* Na HEPES pH 7.0) and reservoir solution (0.1 *M* Na HEPES pH 7.0, 0.6–0.8 *M* sodium/potassium tartrate) against 1 ml reservoir solution. Crystals appeared overnight and grew to their final size (0.2 ± 0.1 × 0.2 ± 0.1 × 0.5 ± 0.1 mm) within a couple of days. Cryoprotection was achieved by supplementing the reservoir solution with 20%(*v*/*v*) ethylene glycol. Crystals were briefly rinsed in this solution before cryocooling in liquid nitrogen. Microcrystals were prepared by rapidly mixing 400 µl 88 mg ml^−1^ thaumatin in 100 m*M* Na HEPES pH 7.0, 400 µl 1.6 *M* sodium/potassium tartrate and 20 µl submicrometre-sized thaumatin seed crystals (details to be published elsewhere). Microcrystals (∼3 × 3 × 5 µm) appeared overnight. The microcrystalline slurry was centrifuged and washed twice with a buffer consisting of 0.1 *M* Na HEPES pH 7.0, 0.8 *M* sodium/potassium tartrate. The preparation of lysozyme–Gd microcrystals is described in Barends *et al.* (2014 ▸).

### 2.2. Comparative synchrotron data collection   

A high-resolution sulfur SAD data set was collected from a single thaumatin crystal on beamline X06DA (PXIII) of the Swiss Light Source (SLS), Villigen, Switzerland. The diamond-shaped crystal (∼0.3 × 0.3 × 0.6 mm) was kept at 100 K during data collection using X-rays of 6 keV photon energy (2.066 Å wavelength). The photon flux was 2 × 10^9^ photons s^−1^ in 90 × 50 µm. Data were collected using three different χ settings differing by 15° using the Parallel Robotics Inspired Goniometer (PRIGo) goniometer (Waltersperger *et al.*, 2015 ▸).

### 2.3. Collection of SFX diffraction data   

SFX measurements on thaumatin microcrystals were performed in July 2014 (proposal LD23) at the LCLS, Menlo Park, California, USA. Shortly before SFX data collection, the thaumatin microcrystal slurry was filtered through a tandem set of 20 and 10 µm stainless-steel filters. An inline stainless-steel 20 µm filter was used during injection. Crystal settling was prevented by the use of an anti-settling sample-delivery instrument (Lomb *et al.*, 2012 ▸) equipped with syringe-temperature control (set to 20°C) for injection. A gas dynamic virtual nozzle liquid jet injector (Weierstall *et al.*, 2012 ▸) was used to deliver the microcrystals in an ∼5 µm diameter liquid jet into the microfocus vacuum chamber of the CXI instrument (Liang *et al.*, 2015 ▸) using an HPLC system (Shimadzu Biotech, Duisburg, Germany). After every 10 min of microcrystal injection the HPLC and injector lines were washed for about 2 min with ultrapure water to reduce the risk of clogging. The sample flow rate was 30 µl min^−1^.

Single-shot diffraction images were recorded at a repetition rate of 120 Hz using a Cornell–SLAC pixel-array detector (CSPAD; Hart *et al.*, 2012 ▸). The detector was placed at a distance of approximately 64 mm from the interaction region. The X-ray pulse energy was 4.3 mJ on average, the photon energy was 6 keV (2.066 Å) and the duration of the pulses was estimated to be ∼30 fs from the measured duration of the electron beam (Behrens *et al.*, 2014 ▸). The beamline transmission was ∼50%. The intensity of the X-ray beam was additionally reduced by inserting solid attenuators into the nonfocused beam (resulting in a transmission of ∼14%) to avoid collecting a significant fraction of saturated reflections and to reduce the risk of damaging the detector by bright reflections from inadvertent salt or ice crystals. The online monitoring tool from the *CASS* software suite (Foucar *et al.*, 2012 ▸) was used during data collection to calculate the online hit rate and the fraction of saturated spots, thereby guiding the positioning of the liquid jet and the transmission setting, respectively, and to display an accumulated virtual powder pattern. Bragg spots extended to the edge of the detector, but geometric limitations in the experimental setup prevented the recording of diffraction data with a resolution of higher than 2.1 Å. The collection of the lysozyme Gd-derivative SFX diffraction data has been described by Barends *et al.* (2014 ▸). The raw SFX diffraction data for the lysozyme Gd derivative are available in the CXIDB database (data set ID 22; Maia, 2012 ▸).

### 2.4. Data processing, phasing and refinement   

The raw XTC data files of thaumatin and lysozyme Gd-derivative microcrystals from the CXI instrument were analysed using the *CASS* software suite (Foucar *et al.*, 2012 ▸). Diffraction snapshots identified as crystal hits were saved in HDF5 format for indexing and Monte Carlo averaging using *CrystFEL* v.0.6.1 (White *et al.*, 2012 ▸). The multi-segment detector geometry was optimized using a Python script that translates and rotates individual tiles of the detector to minimize the distance between the predicted and observed spot locations. The value for the distance between the interaction region (sample) and the detector was refined by manually adapting the distance to minimize the standard deviation of the distribution of unit-cell lengths. Using the *scale* option in *CrystFEL*, the integrated intensities from each diffraction image were multiplied by a scale factor *s*, defined as 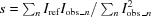, where *I*_ref_ is the mean intensity of a reflection obtained from the first merging pass over all indexed images and *I*_obs_ is the intensity of a reflection in a single image and the summation is over all *n* reflections (*hkl*s) in an image (see Supplementary Fig. S1 for the lysozyme Gd derivative). Scaled intensities were then averaged following the Monte Carlo approach [introduced by Kirian *et al.* (2011 ▸) and implemented in *CrystFEL*], exported to XDS_ASCII format, scaled (with the removal of Wilson plot outliers) using *XSCALE* and converted to structure-factor amplitudes in MTZ and SHELX formats using *XDSCONV* from the *XDS* software package (Kabsch, 2010 ▸). For the lysozyme Gd-derivative data sets, initial phases from the Gd-atom substructure were obtained in 1000 trials of *SHELXD* (Sheldrick, 2010 ▸) using a resolution cutoff of 2.3 Å. Phases were further improved and the resolution was extended to 1.8 Å using *SHELXE* with an initial search for secondary structure. Four runs of 20 cycles of density modification and three cycles of autotracing were used. These improved phases were used for final model building using the *AutoBuild* routine from *PHENIX* (Adams *et al.*, 2010 ▸).

Sulfur phasing of SFX diffraction data from thaumatin microcrystals was performed similarly, using 10 000 trials of *SHELXD* at a resolution cutoff of 3.5 Å. Phases from the initial S-atom substructure were further refined and an initial model was autobuilt using *autoSHARP*. Additionally, phasing and automated model-building tools implemented in *autoSHARP* (Vonrhein *et al.*, 2007 ▸) and *PHENIX AutoSol* were used. Final model building and refinement was performed in iterative cycles of manual model improvement in *Coot* (Emsley & Cowtan, 2004 ▸) and refinement using *PHENIX*. The model quality was checked using *MolProbity* (Chen *et al.*, 2010 ▸) and *PROCHECK* (Laskowski *et al.*, 1993 ▸).

The synchrotron SAD data were processed with *XDS* (Kabsch, 2010 ▸). Phasing and refinement were performed as described above.

## 3. Results   

### 3.1. Lysozyme Gd-derivative SAD phasing and automatic model building   

**Figure 1:**
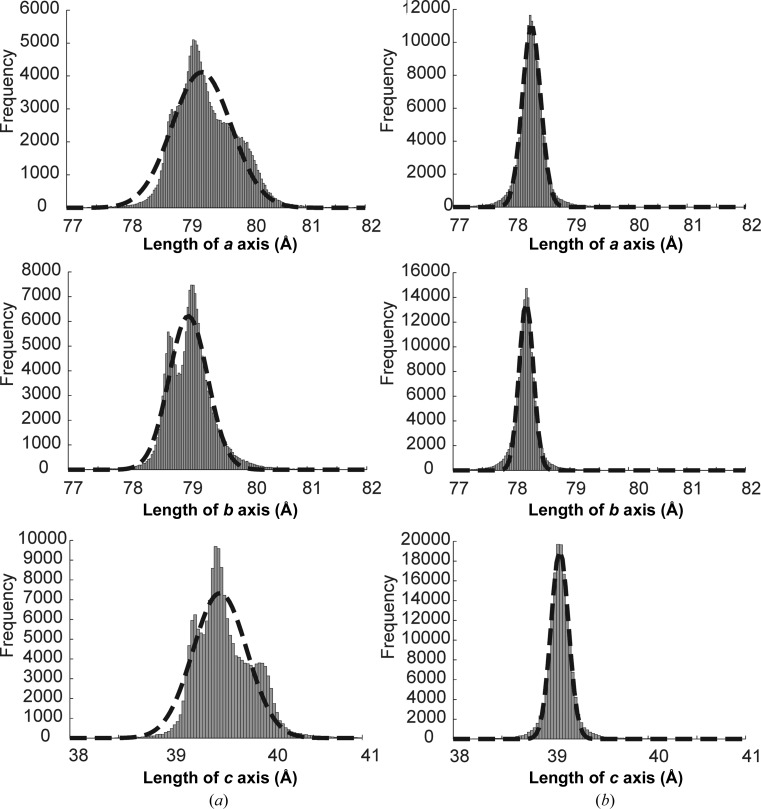
Histograms showing the distribution of the unit-cell parameters *a*, *b* and *c* (*a*) before and (*b*) after optimization of the detector distance using lysozyme–gadolinium images. The dashed lines are Gaussian fits to the individual histograms. Before optimization, broad, multimodal distributions are observed. After optimization, much narrower, single-Gaussian-like distributions are obtained. It is important to note that a lower number of images could be indexed after detector-distance optimization (171 909 before optimization and 155 605 after optimization), suggesting that the highest number of indexed images may not necessarily result in the best quality of the data set.

The raw XTC files containing lysozyme Gd-derivative diffraction images were downloaded from CXIDB (Maia, 2012 ▸) and re-analysed using the *CASS* software suite (Foucar *et al.*, 2012 ▸). *CASS* found 423 647 crystal hits from 1 926 034 recorded detector frames (22.0% hit rate) and *CrystFEL* v.0.6.1 (White *et al.*, 2012 ▸) indexed 171 909 hits (40.6% indexing rate). Adjusting the detector geometry improved the indexing rate and the overall statistics of the data set [from 131 056 indexed images before geometry optimization (30.9% indexing rate) to 171 909 indexed images after geometry optimization (40.6% indexing rate)]. The same sample-to-detector distance (112 mm) as reported by Barends *et al.* (2014 ▸) was used, yielding unit-cell parameters *a* = 79.34, *b* = 79.05, *c* = 39.59 Å, α = β = γ = 90°. A histogram of the unit-cell parameters over all indexed images (Fig. 1 ▸ *a*) did not display the expected single-Gaussian-like distribution^1^ but rather appeared to be a mixture of at least three Gaussian-like distributions for the unit-cell lengths *a* and *c* and a mixture of two Gaussian-like distributions for the unit-cell length *b*. One possible explanation is that the injected microcrystals contained subpopulations of non-isomorphous crystals that could be split into at least two groups with a significantly different *b* axis, separated by a minimum at around 78.8 Å, as seen in Fig. 1 ▸(*a*). To test this hypothesis, diffraction images of crystals with an apparent *b* axis smaller or larger than 78.8 Å were processed separately. However, this procedure did not result in the anticipated data sets with single-Gaussian-shaped histograms of the unit-cell length distributions, not even for the *b* axis. Instead, the histograms maintained the shapes of their original multimodal distributions (not shown). This suggested that the sample-to-detector distance was not identical for all diffraction images merged into the SFX data set and that this difference contributes to the broad multimodal distributions of the unit-cell lengths.

#### 3.1.1. Optimization of the detector distance   

In order to determine whether the multimodal distribution of the unit-cell parameters could be owing to variations in the sample-to-detector distance in different portions of the data set, we tabulated the indexed images with a *b* axis smaller or larger than 78.8 Å as a function of the run number. Supplementary Fig. S2 shows that more images were indexed with the *b*-axis length larger than 78.8 Å between runs 1 and 30, while the *b* axis was smaller than 78.8 Å after run 30. This suggests that the sample-to-detector distance had changed between run numbers 30 and 31, and that it was this change in distance that resulted in an apparent change in the unit-cell lengths. The fact that runs 30 and 31 were collected at the end and the beginning of two 12 h shifts of data collection, with 12 h off in between, made this a highly plausible scenario. Therefore, we grouped the diffraction images into three subgroups, called ‘day 1’ (runs 1–8), ‘day 2’ (runs 9–30) and ‘day 3’ (runs 31–40) and re-processed them. For each group the detector distance was changed in steps of first 100 µm and then 20 µm until the distributions of unit-cell lengths resembled single-Gaussian-like distributions (Fig. 1 ▸ *b*). As an internal control, all initial processing steps were performed assuming a triclinic lattice, thereby avoiding any bias during the detector-distance optimization by imposing a tetragonal symmetry. Importantly, the difference between the *a* and *b* unit-cell lengths decreased from 0.3 to 0.1 Å and the difference of the α, β and γ angles from 90° decreased (data not shown), leading to an improved apparent tetragonal symmetry of the metrics. The intensities were then merged into a final data set consisting of 155 605 indexed images. Supplementary Table S1 shows the average unit-cell values for the data sets before and after detector optimization and also the value of the detector distance before optimization (112.0 mm) and after optimization (110.75, 110.68 and 111.30 mm for the three shifts, respectively). After optimization, the average unit-cell parameters for the whole data set were *a* = 78.37, *b* = 78.27, *c* = 39.12 Å, α = β = γ = 90°. For further processing, the *a* and *b* unit-cell lengths were set to the average of the two values to obey the tetragonal unit-cell constraints.

#### 3.1.2. Comparison of the data statistics before and after detector-distance optimization   

Supplementary Table S2 shows the overall data-quality statistics of whole data set before and after detector-distance optimization, as well as the statistics of the separately processed individual shifts. Despite a lower number of indexed images in the optimized case, detector-distance optimization noticeably improved statistical quality measures such as *R*_split_, CC_1/2_, CC*, *R*_ano_/*R*_split_, CC_ano_ and the signal-to-noise ratio (SNR) of the entire data set and did so over the full resolution range (20–1.8 Å), notably including the highest resolution shell (1.9–1.8 Å). Comparisons of various statistical data-quality measures for the whole data sets before and after detector-distance optimization are shown in Supplementary Fig. S3 as a function of resolution and in Supplementary Fig. S4 as a function of the number of images. All data-quality measures improved after detector-distance optimization, especially at high resolution.

#### 3.1.3. Determination of the minimal number of images necessary for phasing and complete automatic model building   

The *SHELXC*/*D*/*E* pipeline (Sheldrick, 2010 ▸) as implemented in the *HKL*2*MAP* package (Pape & Schneider, 2004 ▸) was used for SAD phasing using subsets of the whole data sets for lysozyme–Gd consisting of decreasing numbers of indexed images before and after detector optimization. Individual images from each data set were chosen randomly and the number of images used for the whole data set (155 605) was repeatedly divided in half until 9725 images were reached; the data set with the smallest number of images consisted of 7000 randomly chosen indexed images.

To test the influence of the quality of individual images on the merged data, a data set was created by selecting images from the whole data set that have a minimum correlation coefficient (CC_min_) between their integrated intensities and the merged intensities (Supplementary Fig. S5). Using a CC_min_ of ≥ 0.83 resulted in the selection of 7251 images of the whole merged (155 605 images) data set. The overall data statistics for the whole resolution range and for the highest resolution shell for the different data sets after detector-distance optimization are shown in Supplementary Table S3 and are plotted for all resolution shells in Supplementary Fig. S6. As expected, the quality of the statistics and the multiplicity of the measurements decrease with the number of images merged in a data set. The data set containing those 7251 images selected according to their CC_min_ value displayed better statistics than a data set containing a similar number (7000) of randomly selected images; in particular, CC_ano_ was significantly higher at low resolution. Analogous data sets before detector-distance optimization were prepared in a similar way, decreasing the number of all indexed images (171 909) by half until 5414 images were reached. The overall data-set statistics for the whole resolution range for data sets before detector-distance optimization are shown in Supplementary Table S4.

**Figure 2:**
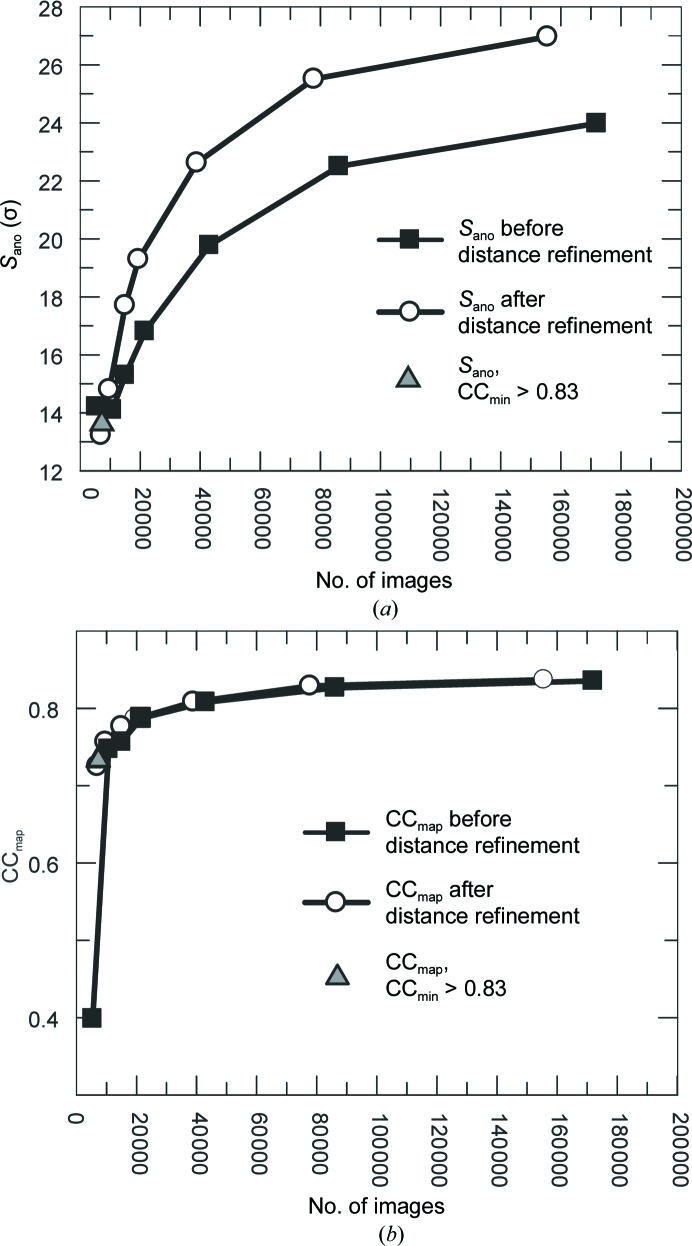
Relationship between the number of images used and (*a*) the anomalous signal strength *S*_ano_ and (*b*) the map quality CC_map_ both before (filled squares) and after (open circles) detector-distance refinement, as well as after the selection of images with a CC_min_ of >0.83 (grey triangle).

Using a resolution cutoff of 2.3 Å and 1000 trials, *SHELXD* was employed to investigate how the number of images in the merged data set influenced determination of the substructure. In each case, the correct substructure consisting of two Gd ions per asymmetric unit was identified as indicated by the two distinct clusters in the plot of CC_all_ (the correlation coefficient between all normalized structure-factor amplitudes *E*_obs_ and *E*_calc_ for each trial) *versus* CC_weak_ (the correlation coefficient between *E*_obs_ and *E*_calc_ for weak reflections, *i.e.* those where *E*_obs_ < 1.5, which *SHELXD* does not use for dual-space cycling; Schneider & Sheldrick, 2002 ▸) (see Supplementary Figs. S7 and S8). The distance between the two clusters decreased when the number of images was reduced. Refinement of phases and density modification assuming a solvent content of 0.43 in *SHELXE* indicated a clear preference for one hand and the correct enantiomorphic space group from the map statistics (connectivity and contrast metrics; Supplementary Tables S5 and S6) for all data sets before and after detector-distance refinement, except for the data set without detector-distance optimization containing the smallest number of images (5414). Subsequently, density-modified maps from *SHELXE* were used in the *PHENIX AutoBuild* routine. For all data sets containing ≥9725 images after detector-distance optimization and ≥10 735 images before detector-distance optimization all 129 residues were built automatically. In contrast, for the data set consisting of 7000 randomly chosen or 7251 selected (CC_min_ ≥ 0.83) images after detector-distance optimization automatic model building resulted in 71 (25 built with the correct sequence) or 89 (74 built with the correct sequence) of 129 residues, respectively. Both models could be completed by a few rounds of manual model building and refinement using *Coot* and *REFMAC*5. Fig. 2 ▸(*a*) shows *S*_ano_ values for lysozyme–Gd data sets as a function of the number of images before and after optimization of the sample-to-detector distance. *S*_ano_ is the average peak height in the anomalous difference Fourier map phased using the model refined against the data set consisting of all indexed images after detector-distance optimization and it can be used to describe the anomalous signal of the data set (Bunkóczi *et al.*, 2015 ▸). Fig. 2 ▸(*b*) shows the CC_map_ values calculated between different maps obtained from *SHELXE* and the final map obtained from the refined model phased using all diffraction images before and after sample-to-detector distance optimization, also as a function of the number of images. It is noticeable that the optimization improved the anomalous signal appreciably. However, the minimal number of images (∼10 000) which resulted in a data set from which a structure could be determined completely automatically is independent of detector-distance optimization.

### 3.2. Thaumatin native S-SAD phasing and automatic model building   

The choice of photon energy for data collection for native SAD phasing is a compromise between the quest for a high anomalous signal of the relatively light atoms (S, P, Ca *etc.*) and thus low photon energy and the limitations set by beamline transmission, detector quantum efficiency, the resolution of the diffraction data and photon absorption, which often benefit from higher photon energy. As the sulfur *K* edge is at 2.47 keV, the collection of anomalous data must be performed at a photon energy far from the edge. Mueller-Dieckmann *et al.* (2005 ▸) showed that for macroscopic crystals the best signal is obtained when collecting sulfur SAD data at 6 keV. Although absorption effects are of no concern for SFX data collection using microcrystals in vacuum, we also chose 6 keV for data collection in order to collect high-resolution data. Nakane *et al.* (2015 ▸) used 7 keV for their lysozyme data collection.

The SFX data set of thaumatin was analysed with *CASS*, which identified 667 504 crystal hits out of 3 729 601 recorded detector frames (17.9% hit rate). The *CrystFEL* software suite v.0.6.1 (White *et al.*, 2012 ▸) was used to index 363 300 hits (54.6% indexing rate) after detector-distance and detector-geometry optimization as described above for the analysis of the lysozyme Gd derivative. The optimized crystal-to-detector distance was 63.35 mm. Supplementary Figs. S9, S10 and S11 show the distributions of unit-cell lengths before detector-distance optimization, after the first step of optimization and after the final third step of detector-distance optimization, respectively. The average unit-cell parameters of the thaumatin microcrystals were *a* = 58.6, *b* = 58.6, *c* = 151.3 Å, α = β = γ = 90°. Thaumatin crystallizes in space group *P*4_1_2_1_2.

Analogously to the case of the lysozyme Gd derivative, we used the full thaumatin data set consisting of 363 300 indexed images to create data sets containing fewer, randomly selected images (200 000, 181 000, 150 000, 125 000 and 100 000). In addition, as described for the lysozyme–Gd data set, we assembled a ‘high-quality’ data set by setting a CC_min_ cutoff of ≥0.72, which resulted in 114 540 images from the complete set (363 300 images). The overall data-quality measures for these data sets (*R*_split_, CC_1/2_, CC*, CC_ano_, *R*_ano_/*R*_split_ and SNR) as a function of resolution as well as their dependence on the number of images are shown in Supplementary Figs. S12 and S13, respectively, and are listed in Supplementary Table S7. The data statistics for the full SFX data set (363 000 images), a reduced data set that still allowed *de novo* phasing (125 000 images) and the synchrotron comparison data set are shown in Table 1 ▸.

**Figure 3:**
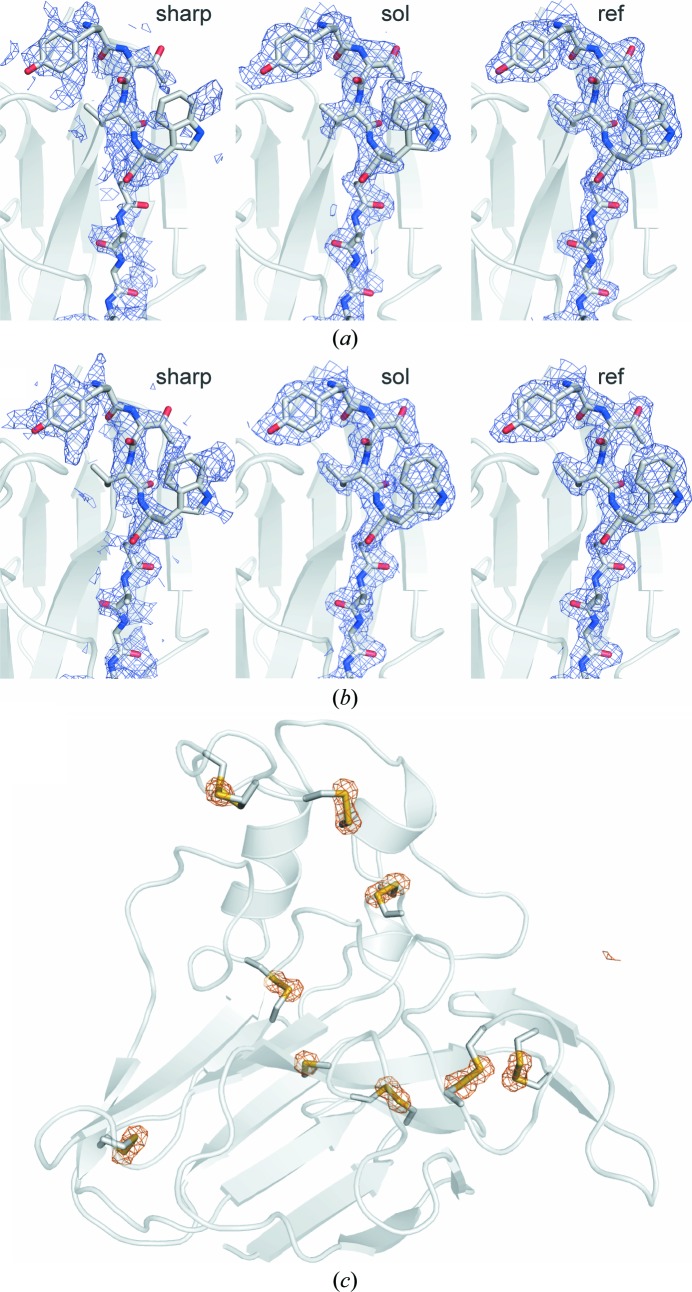
(*a*) Electron-density maps from different stages of the sulfur SAD phasing process for thaumatin using 125 000 SFX images contoured at 1σ. The map calculated from the S-SAD phases calculated by *SHARP* is shown on the left (‘sharp’), the map after solvent flattening in the middle (‘sol’) and the final refined map (2*mF*_o_ − *DF*_c_) on the right (‘ref’). (*b*) As (*a*) but using all (363 300) available images. (*c*) Anomalous difference density map calculated using 125 000 images and phases from the final refined model (shown as a ribbon model; cysteine residues and the single methionine residue are depicted as stick models). The map (orange mesh) is contoured at 5σ.

We first used a conventional crystallographic phasing approach to show that *de novo* S-SAD phasing of SFX data is feasible. Briefly, the substructure of anomalously scattering atoms was identified using *SHELXD*. The correct sites were filtered from the top solutions based on their refined occupancy and cumulative occurrence in the best solutions as judged by their correlation coefficients. Finally, this substructure was used for further phase improvement and automatic building by *autoSHARP*. As an initial proof of concept, a data set consisting of all 363 300 indexed diffraction patterns of thaumatin microcrystals was used for phasing. Since thaumatin contains 16 cysteines and one methionine residue, we searched for 17 potential sites of anomalous scatterers. Furthermore, given the low resolution to which significant anomalous signal was observed (see Supplementary Figs. S12*d*, S12*e* and S14), eight disulfide units were searched for by *SHELXD* using the DSUL option. In this way, we excluded the possibility that disulfide bonds were ignored owing to distance criteria and assumed that all 16 cysteine residues could potentially form disulfide bonds. In fact, *SHELXD* identified a distinct cluster of top solutions (487 solutions with CC_all_ > 30 and CC_weak_ > 9; Supplementary Fig. S15*a*), and the best solution (CC_all_ = 41.31, CC_weak_ = 17.10, Patterson figure of merit PATFOM = 1.39) was used for further phase improvement (see Supplementary Table S8). Although *SHELXD* identified 32 potential sites, a significant decrease in occupancy was observed after the 17th positon. The initial phases determined using this substructure of anomalously scattering atoms were sufficient for automated phase improvement in *autoSHARP* and, after density modification by *SOLOMON*, *ARP*/*wARP* built a model comprising 202 of the 207 residues and 129 water molecules (see Fig. 3 ▸). The refinement converged with *R*_work_ = 19.7% and *R*_free_ = 22.9%. Similarly, 181 650 indexed and scaled diffraction patterns were sufficient to obtain a clearly separated cluster of solutions with high correlation coefficients (Supplementary Fig. S15*b*), although the number of top solutions found in 10 000 trials was significantly lower (119 solutions with CC_all_ ≥ 40 and CC_weak_ ≥ 7). Nevertheless, the best 17 sites of the best solution (CC_all_ = 47.53, CC_weak_ = 10.71, PATFOM = 2.17) were sufficient for subsequent phase improvement (see Supplementary Table S8), density modification and automatic model building using the same approach. Finally, *ARP*/*wARP* converged with an automatically built model comprising 200 of the 207 residues and 117 water molecules with *R*_work_ = 20.4% and *R*_free_ = 23.0% (see Fig. 3 ▸ *a*).

Next, we investigated the number of images required for successful phasing following the same protocol but using a set of only 150 000 randomly selected images for phasing. Although *SHELXD* still found a distinct cluster of top solutions (Supplementary Fig. S15*c*), the number of potentially successful solutions was significantly reduced compared with previous phasing attempts using more diffraction patterns (108 solutions with CC_all_ ≥ 40 and CC_weak_ ≥ 9). Moreover, using the substructure of only the top 17 of the 32 sites that *SHELXD* had identified in the best solution (CC_all_ = 47.0, CC_weak_ = 15.5, PATFOM = 1.39) was insufficient for further phase improvement. Notably, a closer inspection of all 31 sites within this top solution revealed that many of the potential disulfide units were composed of one strong and one weak site, indicating that the chosen top 17 positions are most likely to contain incorrect sites. Thus, the initial phase improvement most likely failed as too many of the correct sites were removed and too many incorrect sites were included. However, when a set of the five top solutions for the sub­structure was transformed to a set of solutions with a common origin and handedness, only 17 of the 31 sites of anomalously scattering atoms that *SHELXD* had identified in each top solution were consistently present in all of the top five solutions, although some of the common sites had low occupancy. Notably, a few sites were found to be single S-atom sites in one solution but were identified as potential disulfides in the other trials. To exclude any bias from prior knowledge of the structure, we also modelled these sites as potential disulfides and assumed that incorrect sites in this filtered substructure will be removed automatically by *autoSHARP*. Indeed, this filtered substructure of anomalously scattering atoms was sufficient for phase improvement (see Supplementary Table S8) and density modification. Finally, *ARP*/*wARP* converged with an automatically built model comprising 202 of the 207 residues and 114 water molecules with *R*_work_ = 19.9% and *R*_free_ = 22.4%.

In order to establish whether a smaller number of diffraction images would also suffice for this conventional S-SAD phasing approach, we reduced the number of indexed images to 125 000. In this attempt, the identification of a potential substructure solution using *SHELXD* became ambiguous and only a limited number of trials were found to be distinct from the rest of the 10 000 trials (ten solutions with CC_all_ ≥ 30 and CC_weak_ ≥ 9; see Supplementary Fig. S15*d*). Nevertheless, once the top five solutions had been transformed to a common origin and handedness, a significant number of sites superimposed perfectly. However, in contrast to phasing attempts using 150 000 images, not all anomalously scattering atom sites were found in all top solutions. Thus, we decided to include all sites that were observed at least three times in a filtered substructure, irrespective of whether they were potential disulfides or single positions, and removed all others. Furthermore, each atom site that was found as a potential disulfide in at least one single solution was modelled as such. Using this method, we identified 22 potential atom sites, ignoring the fact that at least five of them were incorrect. Although we used a rather imperfect substructure model for further phase improvement (see Supplementary Table S8) and density modification in *autoSHARP*, the *ARP*/*wARP* procedure built a model comprising 202 of the 207 residues and 122 water molecules (see Fig. 3 ▸ *b*) and converged with *R*_work_ = 20.2% and *R*_free_ = 22.9%. A comparison of the initial filtered-atom substructure and the final substructure after *autoSHARP* refinement revealed that all false positives were removed except for a single incorrect site, which also had the lowest occupancy.

However, when only 100 000 diffraction patterns were used *SHELXD* did not identify any significantly outstanding solutions among 500 000 trials using data up to 2.03 Å resolution (Supplementary Fig. S15*e*). Thus, filtering a correct solution by superposition of the best solutions was not possible and our S-SAD attempt failed. We next tested whether this was owing to the inability to identify a sufficiently correct substructure of anomalous scatterers, or whether the noise in the anomalous signal within this set of structure-factor amplitudes was too high for successful phasing. The latter is certainly the case, as no interpretable electron density could be obtained when the filtered but slightly incorrect substructure solution obtained from 150 000 images was used for phase improvement, density modification and model building by *autoSHARP*. Additionally, inspection of the electron-density map revealed that manual model building would not be possible for this density either. In contrast, if the exact substructure obtained from scaling all diffraction patterns was used for phasing, *autoSHARP* was able to automatically build an almost complete model (202 of 207 residues and 116 water molecules; *R*_work_ = 19.7% and *R*_free_ = 22.3%) comparable to what could be achieved in all attempts using more than 100 000 diffraction patterns. Apparently, using excellent sites was sufficient to compensate for the increased noise in the set of structure-factor amplitudes of the 100 000-image data set. Along the same lines, we were able to phase the ‘high-quality’ data set (114 540 images having a CC_min_ of ≥ 0.72 relative to the entire data set). *SHELXD* found a distinct cluster of top solutions (Supplementary Fig. S16); 124 solutions showed CC_all_ ≥ 35 and CC_weak_ ≥ 10. The data set could be phased (using *Parrot*) and built (using *Buccaneer*) in *autoSHARP* using a resolution limit of 2.9 Å, yielding a model consisting of 195 residues, of which 165 were sequenced correctly.

## 4. Discussion   

SFX is an emerging technique for radiation-damage-free data acquisition from systems that are challenging to study at synchrotron sources. Despite a number of groundbreaking successes relying on available structural information for phasing, *de novo* structure determination has been scarce and limited so far to well characterized model systems. In part, this is owing to the need for benchmarking when developing new approaches and the requirement for relatively large amounts of well diffracting crystals. Moreover, the use of model systems has the undeniable advantage of knowing the answer, allowing one to establish good-practice procedures. Since the use of SFX as a general tool will depend critically on the ability to carry out *de novo* phasing of unknown structures, this is an essential step in the development of the technique.

SFX data collection is characterized by fluctuations in many experimental parameters, including crystal size and quality, photon energy and spectral distribution, and pulse energy. Moreover, data-collection parameters such as the sample-to-detector distance, wavelength and direct-beam position that are essential for data-analysis programs and are trivial to obtain at synchrotron beamlines are often not known accurately. Together, these issues greatly complicate indexing and the derivation of unit-cell parameters and crystal symmetry; particularly since only a single diffraction image with partial reflections can be obtained from each crystal. It is thus common practice to use the available sample-to-detector distance as a starting point and to refine it by optimizing the fraction of indexed patterns (indexing rate; see, for example, Hattne *et al.*, 2014 ▸). Histograms of unit-cell parameter distributions are plotted and if they agree with the expected values then the detector distance is fixed for the rest of the data collection. Using the sensitive anomalous difference signal from a gadolinium derivative as well as that from endogenous sulfur, we show here that this approach is inappropriate for data analysis with *CrystFEL* and probably likewise with other programs. Careful analysis of unit-cell parameter distributions enabled sample-to-detector optimization after experimental changes (nozzle change, injector removal for cleaning or sample top-up *etc.*), which resulted in fewer indexed images compared with a global optimization of the indexing rate. Despite containing fewer images, these data are, however, of significantly higher quality, suggesting that global optimization of indexing rates can come at the cost of including a significant fraction of misindexed images. Hence, it appears that the accuracy of XFEL data is currently not only limited by intrinsic fluctuations inherent to XFEL data collection, but is also compounded by non-optimized analysis programs that cannot address these fluctuations in combination with issues such as the varying spectral distribution of the XFEL beam, partiality of reflections and complications of post-refinement. This makes it difficult to obtain the highly accurate structure-factor amplitudes required for *de novo* phasing.

Indeed, the few available examples of *de novo* phasing of SFX data show unequivocally that this is still far from trivial. The first report (SAD phasing of lysozyme with two Gd atoms; Barends *et al.*, 2014 ▸) required 60 000 indexed images out of almost 200 000 diffraction patterns identified as crystal hits at the time. These ∼200 000 diffraction patterns in turn represented only a fraction of the 2.4 million images that were collected in total. At an FEL repetition rate of 120 Hz and an injection flow rate of 30 µl min^−1^, around 10 ml of a highly concentrated crystal suspension was required for these measurements. In fact, this estimate neglects the overhead owing to injection nozzle changes *etc.*, so that the true sample consumption was considerably higher. Nakane *et al.* (2015 ▸) make a similar calculation for their native sulfur SAD phasing of lysozyme, which used a high-viscosity injection method that requires much lower flow rates. However, despite the fact that their estimate neglected overhead, they also arrived at the conclusion that *de novo* native SAD phasing of SFX data is highly costly in terms of sample consumption, requiring significantly more crystalline material than is required at synchrotron sources. Thus, to make *de novo* phasing of SFX data feasible for the study of challenging targets which are typically difficult to purify and crystallize, it is crucial that the number of images required for successful phasing be reduced as far as possible.

It is for this reason that we embarked on our systematic study of the factors that affect SFX data phasing, using model systems that are trivial to phase when the data are collected at a synchrotron or home source.

Revisiting the lysozyme–gadolinium data used in Barends *et al.* (2014 ▸) revealed the unexpected multimodal unit-cell distributions shown in Fig. 1 ▸. While it appears that in this case these were caused by differences in the sample-to-detector distance between runs, rather than the occurrence of populations of non-isomorphous crystals, non-isomorphism may still be a factor in other systems and should be checked for in every individual case.

The refined sample-to-detector distance differed by ∼600 µm between days 2 and 3, which is entirely within the expected limit for the experimental setup. However, it cannot be excluded that other, as yet unidentified, factors contribute to the apparent differences in sample-to-detector distance.

Notwithstanding these considerations, optimizing the detector distance on a shift-by-shift basis to enforce unimodal distributions of the unit-cell parameters improved the data-quality metrics *R*_split_, CC_1/2_ and CC*, as well as *R*_ano_/*R*_split_ and CC_ano_, which indicate the strength of the anomalous signal, even though the number of indexed images decreased slightly. We therefore continued our investigation with data processed with detector distances optimized as described. We then attempted to decrease the number of images required for phasing by scaling individual observations of intensities as described above. For the lysozyme Gd-derivative data set, which has a very strong anomalous signal, about 7000 images were sufficient for successful phasing regardless of whether the images were chosen randomly or selected based on their correlation coefficient with the averaged intensities (CC_min_ ≥ 0.83). However, with this comparatively low number of images we did observe a difference in the quality of the phases that were obtained, as shown by the fact that about three times more residues could be automatically assigned to the sequence in the map from the selected images. Fully automated building of the structure with only 10 000 indexed images constitutes a major improvement over the 60 000 images that were required only two years ago.

It is worthwhile noting that the lysozyme Gd-derivative system has a very simple substructure consisting of just two extremely strong anomalously scattering atoms per asymmetric unit. Finding this substructure, as also noted by Barends *et al.* (2014 ▸), is comparatively easy and could be performed with only 10 000 images even at the time of that publication. Here, a large number of images was required for the subsequent phasing step. The only observable effect of reducing the number of images on the substructure search itself in the current study was a decreased apparent distance between the Gd atoms. This may be explained by a reduction of the anomalous signal at high resolution causing the two atoms, which are only ∼6 Å apart, to become progressively more difficult to distinguish. We anticipated that it should be possible to judge the necessary number of images for substructure determination from the number of images where an obvious change in the appearance of the calculated anomalous difference Patterson map occurs. Indeed, there were still very strong peaks in the Patterson map calculated using 7000 images; the substructure could be found easily and the protein structure was phased and built automatically to an extent which could be corrected and finished by manual model building. In contrast, when using a data set containing 5000 images the peaks were very broad, barely extending above the noise. The substructure search was not straightforward, and phasing the protein structure and autobuilding were unsuccessful (data not shown).

We naively assumed that we could use this approach for the thaumatin data as well, using a synchrotron reference data set collected at the same photon energy as used for the SFX data for comparison. The anomalous signal is extremely high and phasing worked fully automatically. The anomalous difference Patterson map from this data is shown in Supplementary Fig. S17(*a*). Using all 363 300 available thaumatin images resulted in a Patterson map (Supplementary Fig. S17*b*) that is more similar to the Patterson map calculated from the reference synchrotron data set than the Patterson map calculated from 125 000 images (Supplementary Fig. S17*c*). When compared with the noise-free Patterson map calculated from *F*_calc_ obtained from the final model (Supplementary Fig. S17*d*), the synchrotron Patterson map and the Patterson map obtained from 363 300 thaumatin images are similar, whereas the Patterson map obtained from 125 000 thaumatin images differs. The reason for the difficulty in assigning heavy-atom positions in the Patterson maps (Supplementary Fig. S17) is that thaumatin contains 17 weakly scattering atoms. For a substructure of 17 weakly scattering atoms, one would expect as many as 272 unique peaks in an anomalous difference Patterson map, making it difficult to interpret. Added to this is the fact that at resolutions worse than 2 Å the S atoms in the disulfides will appear as broad superatoms rather than as sharp point scatterers, compounding the already appreciable peak broadening that complicates every Patterson map. Given this situation, it is not surprising that solving the substructure is challenging, in particular when the number of images was reduced to only 125 000.

In this case, the anomalous signal was reasonable to 3.5 Å resolution and a number of potential solutions needed to be superimposed to build up a consistent ‘filtered’ substructure of common S-atom sites. The difficulty in obtaining a sufficiently accurate substructure in this case is also illustrated by the fact that a substructure solved from 150 000 images was not sufficiently accurate to allow the phasing of a data set from 100 000 images, whereas a substructure solved from all 363 300 images did suffice, even if a 125 000-image substructure could be used to phase a 125 000-image data set. At even lower numbers of images the noise in the structure-factor amplitudes became too large to be overcome even with the most accurate substructure available.

Thus, it appears that incremental yet significant improvements to data quality can be made by three relatively simple procedures: optimizing the detector geometry [other examples of detector-geometry optimization are described in Yefanov *et al.* (2015 ▸) and Hattne *et al.* (2014 ▸)], refining the sample-to-detector distance (possibly as a surrogate for some as yet unidentified factor) and scaling of the individual observations. By combining these steps, a significant reduction in the number of images that are required to solve the lysozyme Gd-derivative structure was obtained. Moreover, using these techniques and adding a peak-filtering procedure to the substructure search allowed us to demonstrate native sulfur SAD phasing of thaumatin with 125 000 images.

Improvements in detectors and processing software should further reduce the number of images required to phase a set of SFX data. However, since only strongly diffracting model systems have been phased with SFX to date, it remains to be seen whether these improvements will suffice to allow the phasing of weaker data of much lower resolution, which in many cases are those of actual scientific interest.

## Supplementary Material

PDB reference: thaumatin, 5fgt

PDB reference: 5fgx

Detailed data analysis and statistics.. DOI: 10.1107/S2052252516002980/mf5014sup1.pdf

## Figures and Tables

**Table 1:** Data-collection, phasing and refinement statistics for the thaumatin S-SAD data sets Values in parentheses are for the outer shell.

	LCLS, 125 000 images	LCLS, all images	Synchrotron data set
Data collection			
Wavelength (Å)	2.066	2.066	2.066
Space group	*P*4_1_2_1_2	*P*4_1_2_1_2	*P*4_1_2_1_2
Unit-cell parameters (Å, °)	*a* = *b* = 58.6, *c* = 151.3, α = β = γ = 90	*a* = *b* = 58.6, *c* = 151.3, α = β = γ = 90	*a* = *b* = 57.7, *c* = 150.0, α = β = γ = 90
No. of indexed images	125000	363300	10845
No. of unique reflections	29350	29350	26126
Resolution range (Å)	20–2.1 (2.2–2.1)	20–2.1 (2.2–2.1)	45.75–2.134 (2.19–2.13)
Completeness (%)	100 (100)	100 (100)	97.0 (61.5)
Multiplicity (SFX)	520.4 (236.1)	1924.9 (688.7)	n.a.
Multiplicity	n.a.	n.a.	28.9 (2.1)
*R*_meas_	n.a.	n.a.	0.041 (0.087)
*R*_split_	0.047 (0.159)	0.027 (0.090)	n.a.
CC_1/2_	0.997 (0.960)	0.999 (0.987)	1.00 (0.991)
CC_ano_	0.128 (0.071)	0.364 (0.097)	0.730 (0.210)
〈*I*/σ(*I*)〉	17.0 (5.9)	29.2 (10.1)	71.3 (8.9)
Refinement			
Resolution range (Å)	20–2.1		45.75–2.134
*R*/*R*_free_	0.156/0.186		0.129/0.177
No. of atoms			
Protein	1557		1567
Ligands	10 [tartaric acid]		10 [tartaric acid]
Water	137		410
Wilson *B* factor (Å^2^)	27.7		17.6
Average *B* factor (Å^2^)			
Protein	27.0		13.9
Ligands	26.3		11.2
Water	34.1		24.9
R.m.s. deviations			
Bonds (Å)	0.007		0.007
Angles (°)	0.987		1.036
Ramachandran plot (% of residues)			
Preferred	97.5		97.0
Allowed	2.5		3.0
Outliers	0.0		0.0
PDB code	5fgt		5fgx

## Footnotes

1 For monochromatic radiation one would expect a Lorentzian (Cauchy)-like distribution. The convolution with the relatively broad photon-energy distribution may result in a Gaussian-like distribution.
