# Supplementary material for: Protein structure determination by single-wavelength anomalous diffraction phasing of X-ray free-electron laser data
Source: IUCrJ. 2016 Mar 9;3(Pt 3):180–91. doi: 10.1107/S2052252516002980 (PMC4856140; doi:10.1107/S2052252516002980)
Supplement: Supplementary file 1 [file m-03-00180-sup1.pdf]

IUCrJ

Volume 3 (2016)

Supporting information for article:

Protein structure determination by single-wavelength anomalous diffraction phasing of X-ray free-electron laser data

Karol Nass, Anton Meinhart, Thomas R. M. Barends, Lutz Foucar, Alexander Gorel, Andrew Aquila, Sabine Botha, R. Bruce Doak, Jason Koglin, Mengning Liang, Robert L. Shoeman, Garth Williams, Sebastien Boutet and Ilme Schlichting

Figure S1 Histogram of the scale factor s values for all single intensity measurements of all reflections from all indexed lysozyme Gd-derivative diffraction patterns after detector distance optimization. The scale factor s is calculated by CrystFEL as follows: $s = \frac{\sum_n I_{ref} I_{obs\_n}}{\sum_n I^2_{obs\_n}}$; where I_{ref} is the mean intensity of a reflection in a single image obtained from the first merging pass over all indexed images and I_{obs} is the intensity of a reflection in a single image and the summation is over all *n* reflections (hkl's) in an image. The scale factor is applied to each individual measurement of a given reflection during the second merging pass. The mean value of the scale factor s across the whole data set is 0.38 ± 0.17. The dashed line is a Gaussian fit to the histogram.

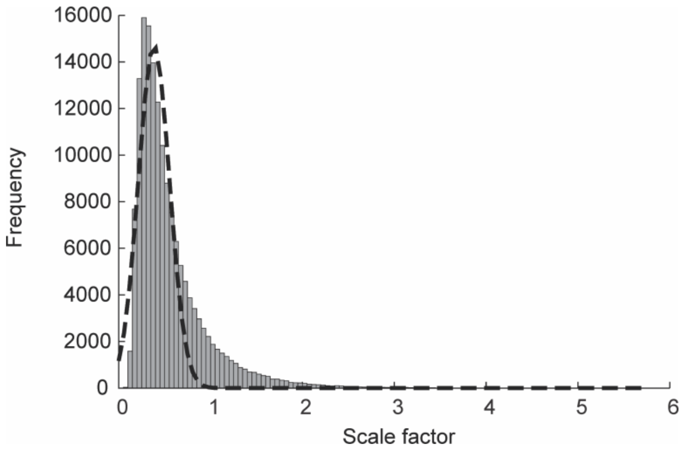

Figure S2 Distribution of the fraction of indexed images with the unit cell *b*-axis smaller (blue line) or larger (red line) than 78.8 Å as a function of the run number. More images indexed with the unit cell *b*-axis larger than 78.8 Å were recorded during runs 1-8 and 19-30 (the red line is above the blue line). In runs 31-40 the opposite is true; more images indexed with the unit cell *b*-axis smaller than 78.8 Å were observed. This suggests that the sample-to-detector distance changed after run 30 (which was not accounted for in the original processing, since all images were processed using the same settings). Such sample-to-detector distance changes could arise due to the disassembly and reassembly of the injector, catcher and lower portion of the nozzle shroud at the end of a shift or even nozzle replacement during a shift after cleaning. Examination of the time stamps in the LCLS electronic logbook for each run revealed that shift changes appeared after the end of run 8 and 30. Thus runs 1-8, 9-30 and 31-40 were recorded on days (or shifts) 1, 2, and 3 respectively.

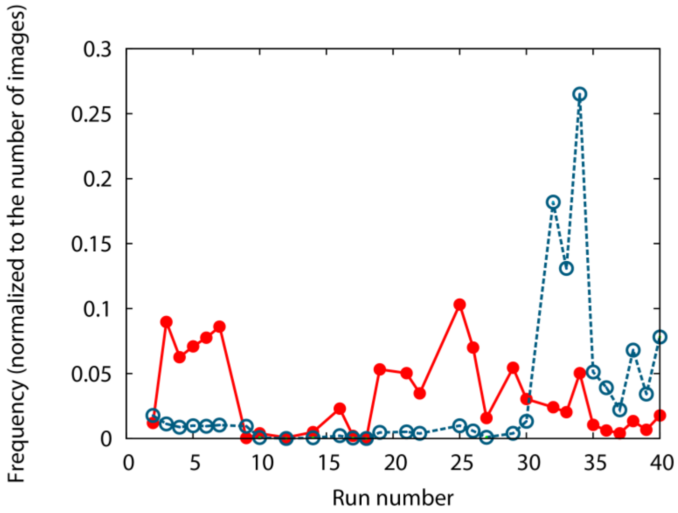

Figure S3 Comparison of the data quality measures of the whole lysozyme Gd-derivative data sets before (solid line, squares) and after (dashed line, circles) detector distance optimization, split into 10 resolution shells containing equal numbers of reflections. The resolution range of the data sets is 20 – 1.8 Å. a) and b) show R_{split} and CC-1/2 values, c) and d) CC* and CC_{ano} values, e) and f) R_{ano}/R_{split} and SNR values. All data quality measures in resolution shells above about 3.5 Å improved slightly after sample-to-detector distance optimization.

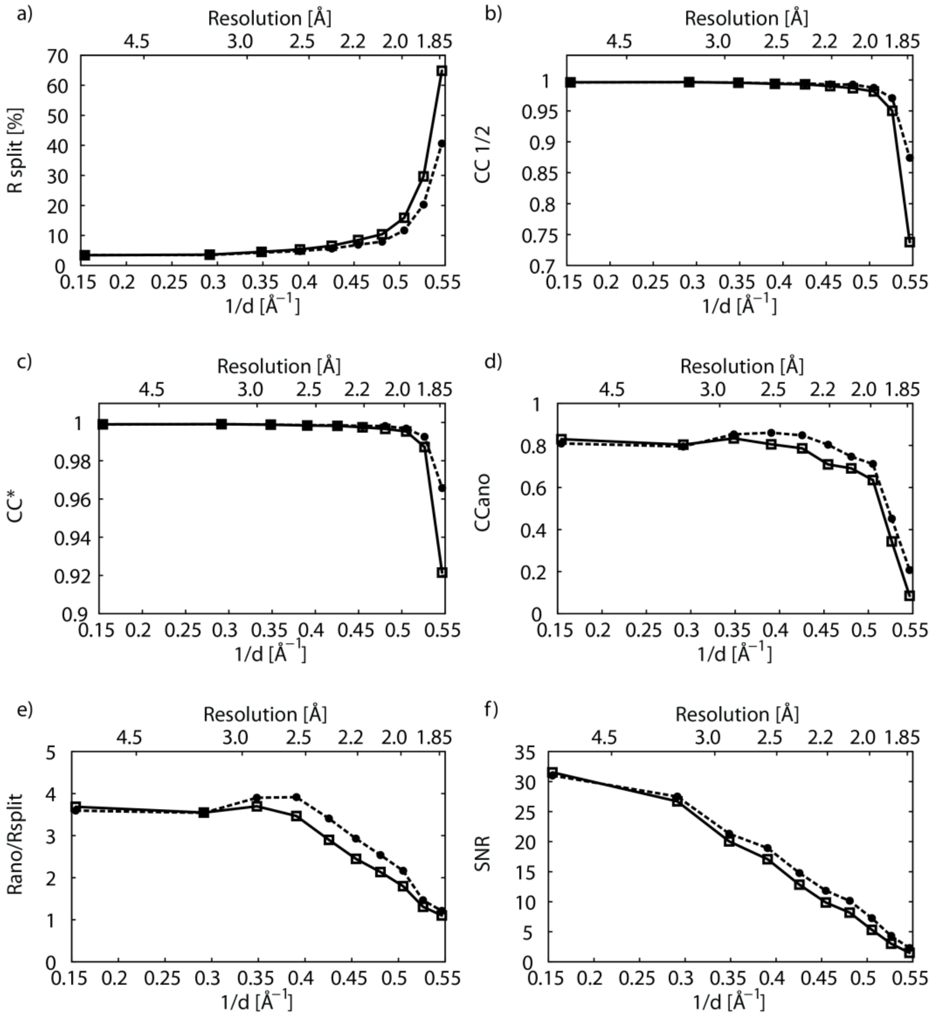

Figure S4 Data quality measures for lysozyme Gd-derivative data sets with increasing number of images in the resolution range 20 – 1.8 Å before (solid line, squares) and after (dashed line, circles) detector distance optimization and for the data set merged with CC_{min}≥0.83 after detector distance optimization (triangle). a) and b) show R_{split} and CC-1/2 values, c) and d) CC* and CC_{ano} values, e) and f) R_{ano}/R_{split} and SNR values. The values for each data point are listed in the Tables S3 and S4. Most of the data quality measures improved after sample-to-detector distance optimization, in particular the signal to noise ratio and R_{split}.

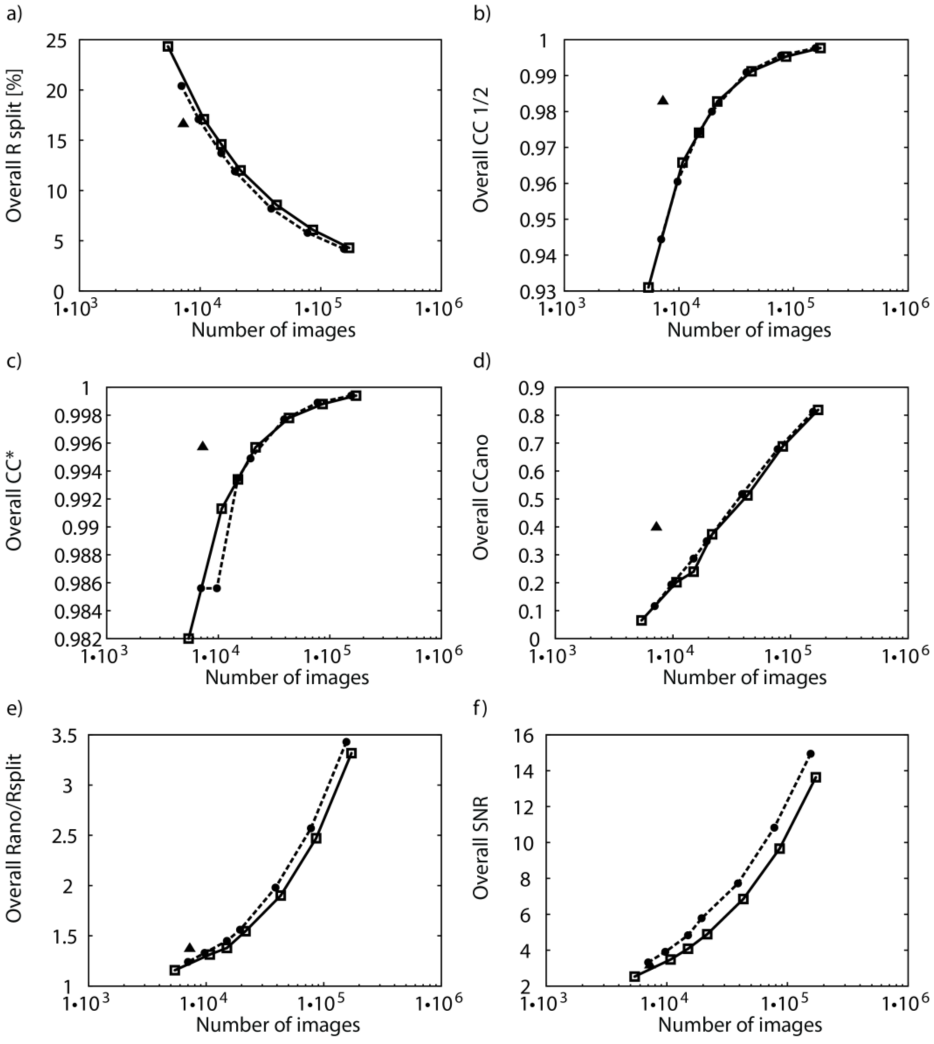

Figure S5 Histogram of the correlation coefficients for all individual intensity measurements of single reflections for all indexed lysozyme Gd-derivative diffraction patterns after detector distance optimization. The CC value is calculated between all reflections in an image and a set of reference reflection intensities obtained from a first pass merging all indexed images. The mean value of the CC across the whole data set is 0.61 ± 0.14. The dashed line is a Gaussian fit to the histogram.

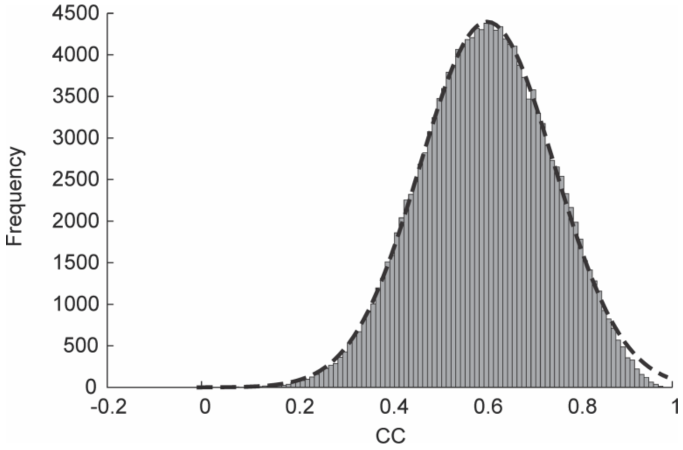

Figure S6 Comparison of data quality measures of lysozyme Gd-derivative data sets after detector distance optimization with decreasing numbers of images. All data subsets span the full resolution range (20 – 1.8 Å), with the number of images, as indicated, being randomly halved from one subset to the next. Insets a) and b) present R_{split} and CC-1/2 values, insets c and d present CC* and CC_{ano} values, e) and f) present R_{ano}/R_{split} and SNR values, and g) presents the redundancy, i.e. the average number of measurements per resolution shell as a function of the resolution and number of indexed images used for Monte Carlo integration. It can be noticed that all data scaling quality measures deteriorate with increasing resolution as well as with a decreasing number of indexed images used for integration. The data set "7k-CC_{min}", which corresponds to the data set merged with $CC_{min} \geq 0.83$ option shows a different trend in its quality measures than all the other data sets. It is better in the low resolution range but deteriorates at higher resolution when compared to the data set that contains a similar number of images merged without the $CC_{min} \geq 0.83$ selection criterion.

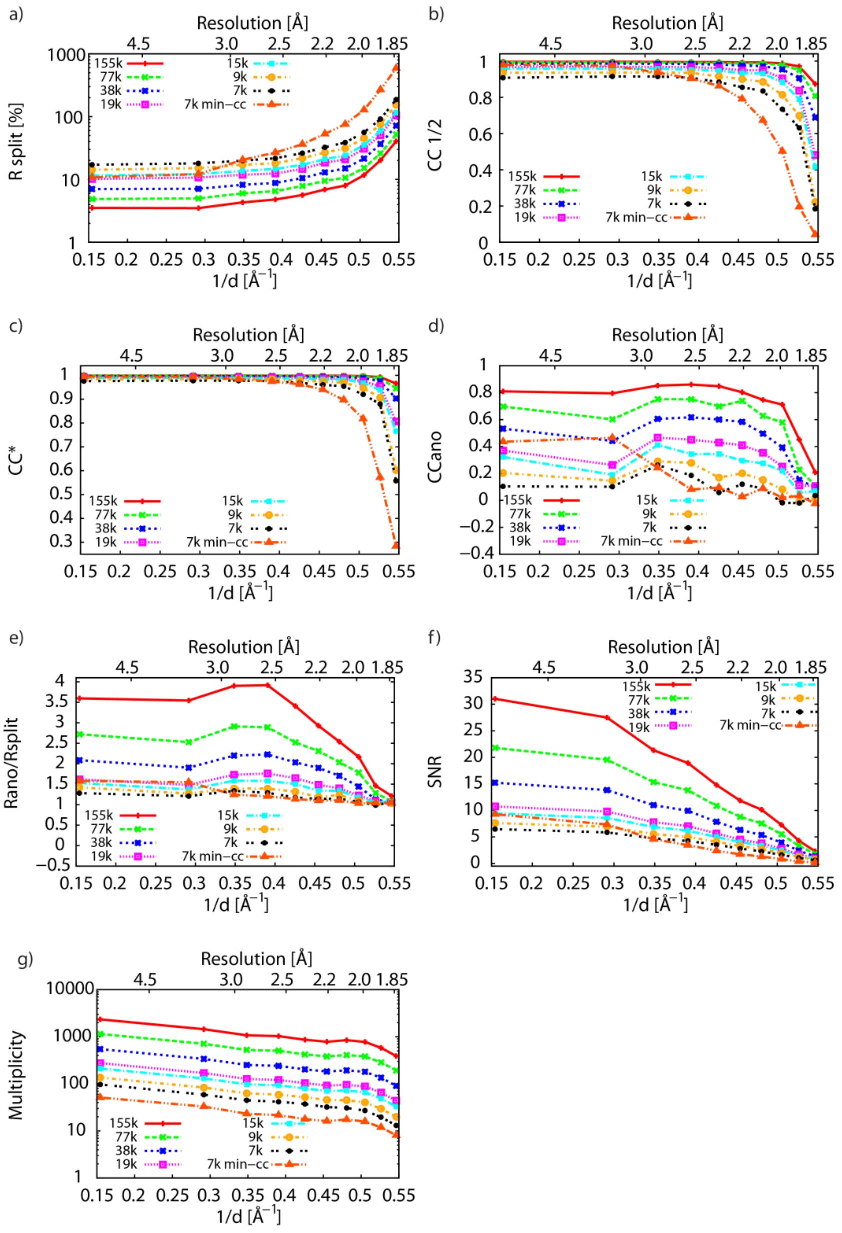

Figure S7 CC_{all} vs CC_{weak} plots from SHELXD for lysozyme Gd-derivative data sets with decreasing number of images before detector distance optimization. 1,000 trials were used to find two Gd sites for each data set at a maximum resolution of 2.3 Å. Distinct clusters with correct substructure solution can be identified for all data sets (red dots). a) shows CC_{all} *vs* CC_{weak} values for all indexed images (171,909), b) for 86,130 indexed images, c) for 43,046 indexed images, d) for 21,613 indexed images, e) for 10,735 indexed images, and f) for 5,414 indexed images.

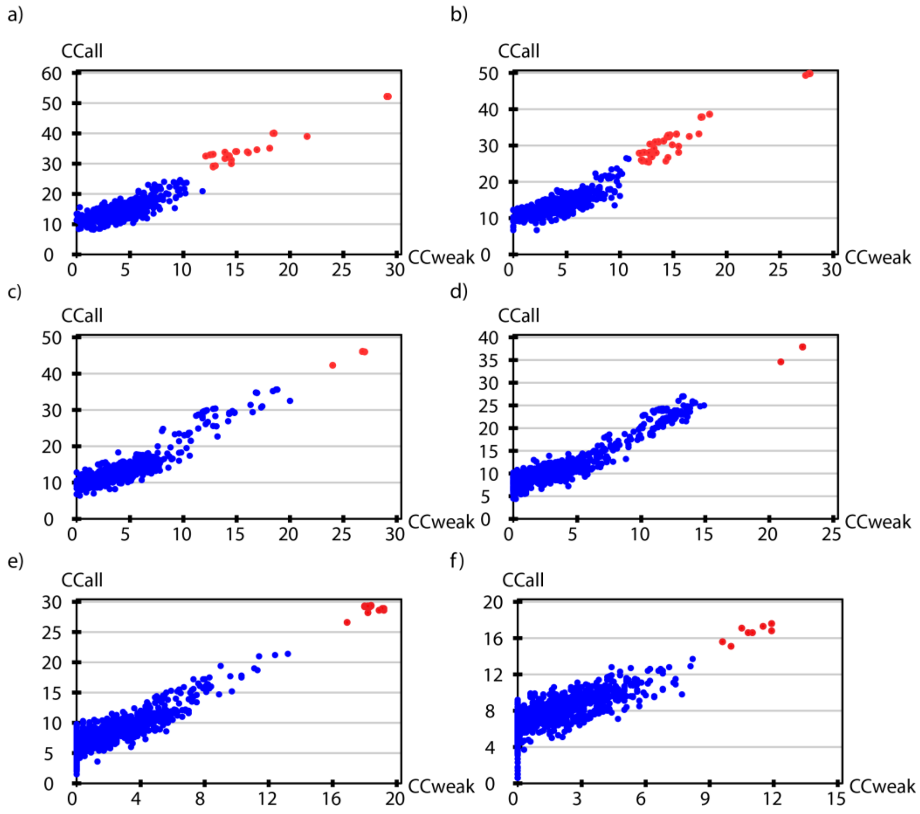

Figure S8 CC_{all} *vs* CC_{weak} plots from SHELXD for lysozyme Gd-derivative data sets with decreasing number of images after detector distance optimization. 1,000 trials were requested to find 2 Gd sites for each data set at a maximum resolution of 2.3 Å. Distinct clusters with correct substructure solution can be identified in all data sets (red dots). a) shows CC_{all} *vs* CC*weak* values for all indexed images (155,605), b) for 77,802 indexed images, c) for 38,901 indexed images, d) for 19,450 indexed images, e) for 15,000 indexed images, f) for 9,725 indexed images, g) for 7,000 indexed images, and h) for 7,251 selected indexed images ($CC_{min} \geq 0.83$).

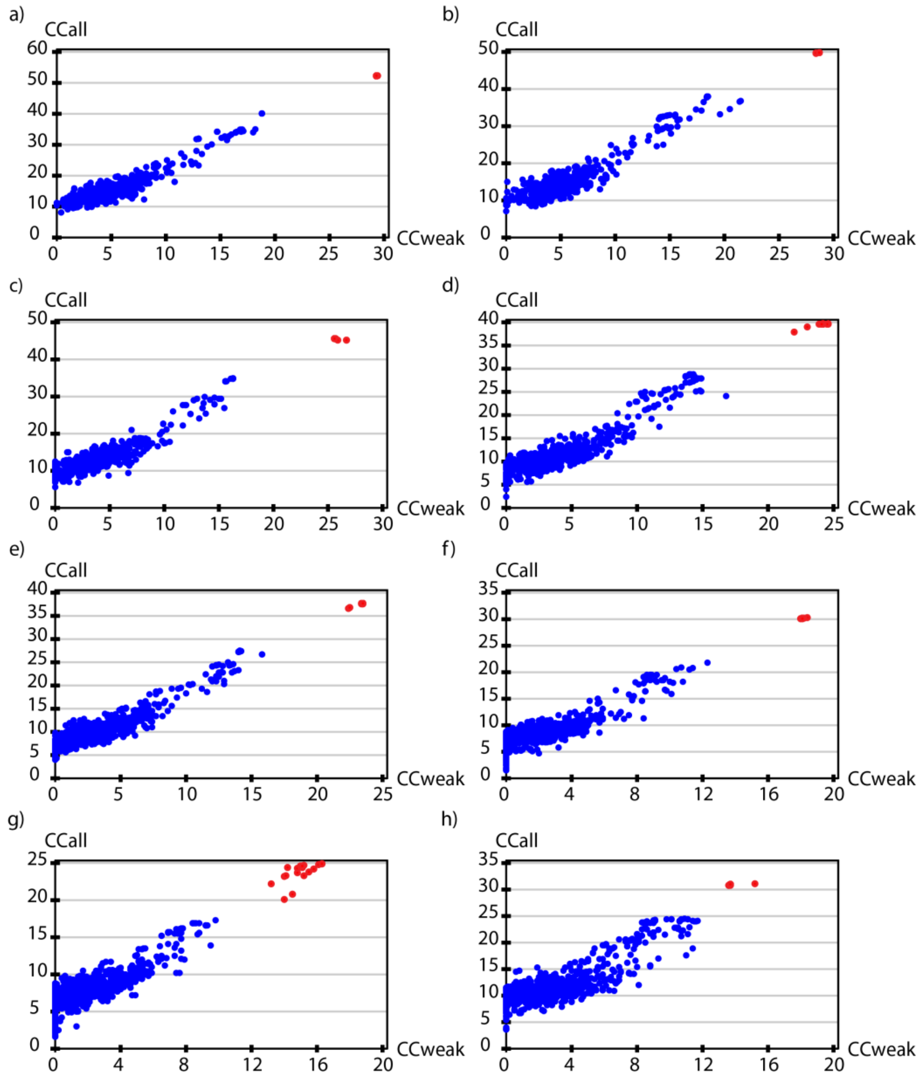

Figure S9 Thaumatin unit cell parameter distribution histograms for the whole data set consisting of 364,782 indexed diffraction patterns before detector distance optimization but after CSPAD geometry optimization. For the initial analysis the nominal detector distance calculated from the encoder values was used (63.852 mm). Judging from the shape of the histograms (skewed distributions, especially pronounced for the *c* axis), the sample-to-detector distance was incorrect. The dashed lines are Gaussian fits to the individual histograms.

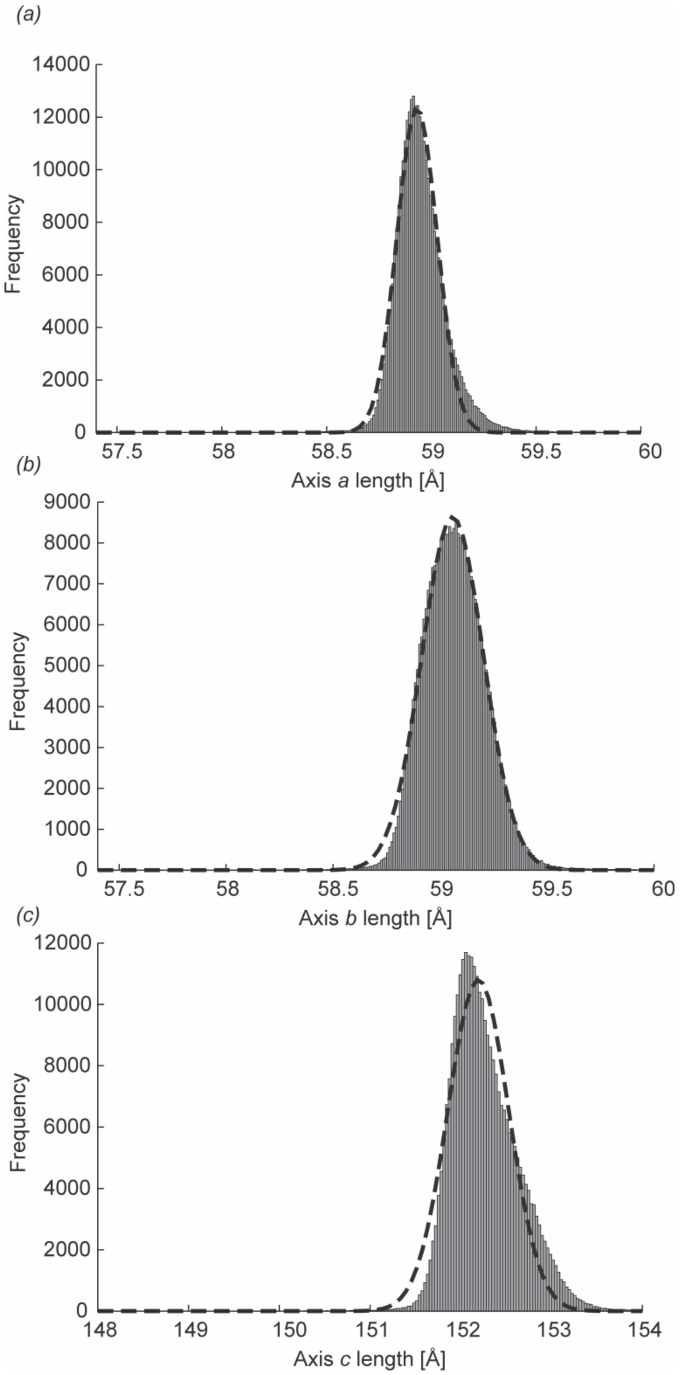

Figure S10 Thaumatin unit cell parameter distribution histograms for the whole data set. The detector distance was changed to 62.852 mm. This was the second optimization step. The dashed lines are Gaussian fits to the individual histograms. Apparently this sample-to-detector distance is also incorrect, judging from the non-Gaussian shape of the distributions (skewed distributions, especially pronounced for the *c* axis).

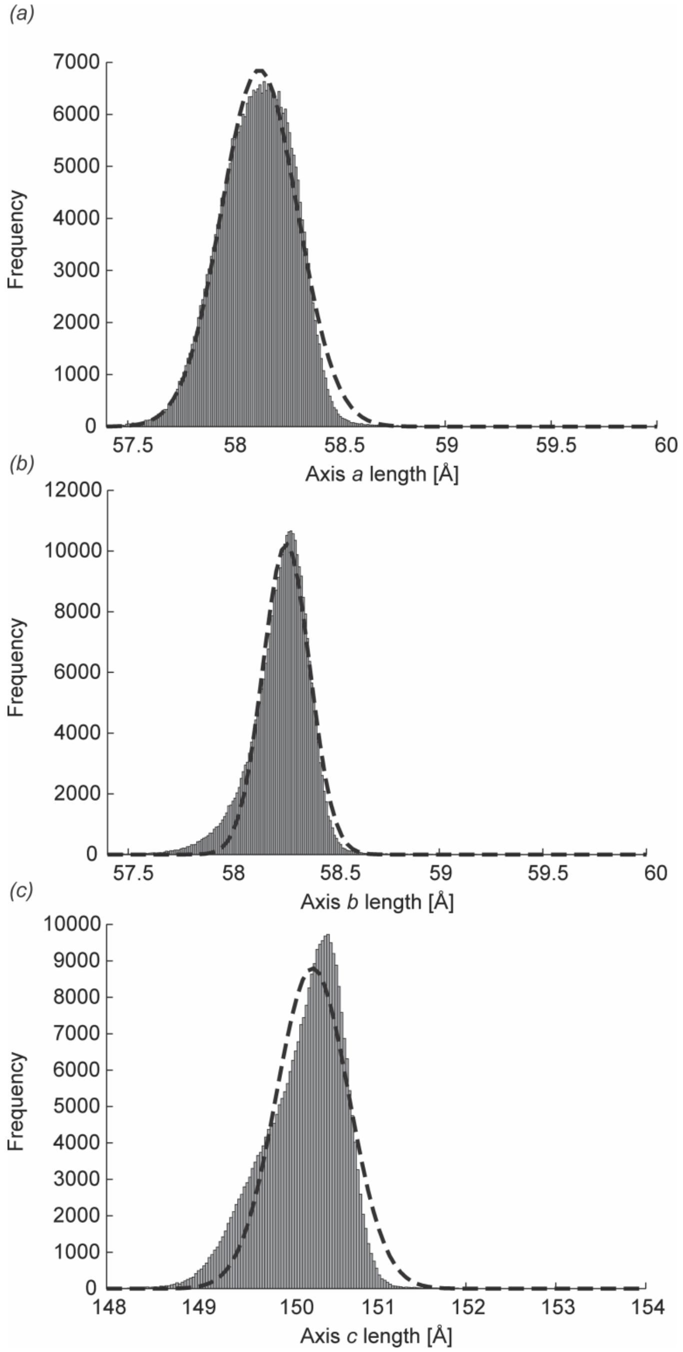

Figure S11 Thaumatin unit cell parameter distribution histograms for the whole data set after the third detector distance optimization step and another CSPAD geometry refinement cycle. The number of indexed images is 363,300. The detector distance refined to 63.352 mm. The dashed lines are Gaussian fits to the individual histograms. The shapes of the distributions are more Gaussian-like than before.

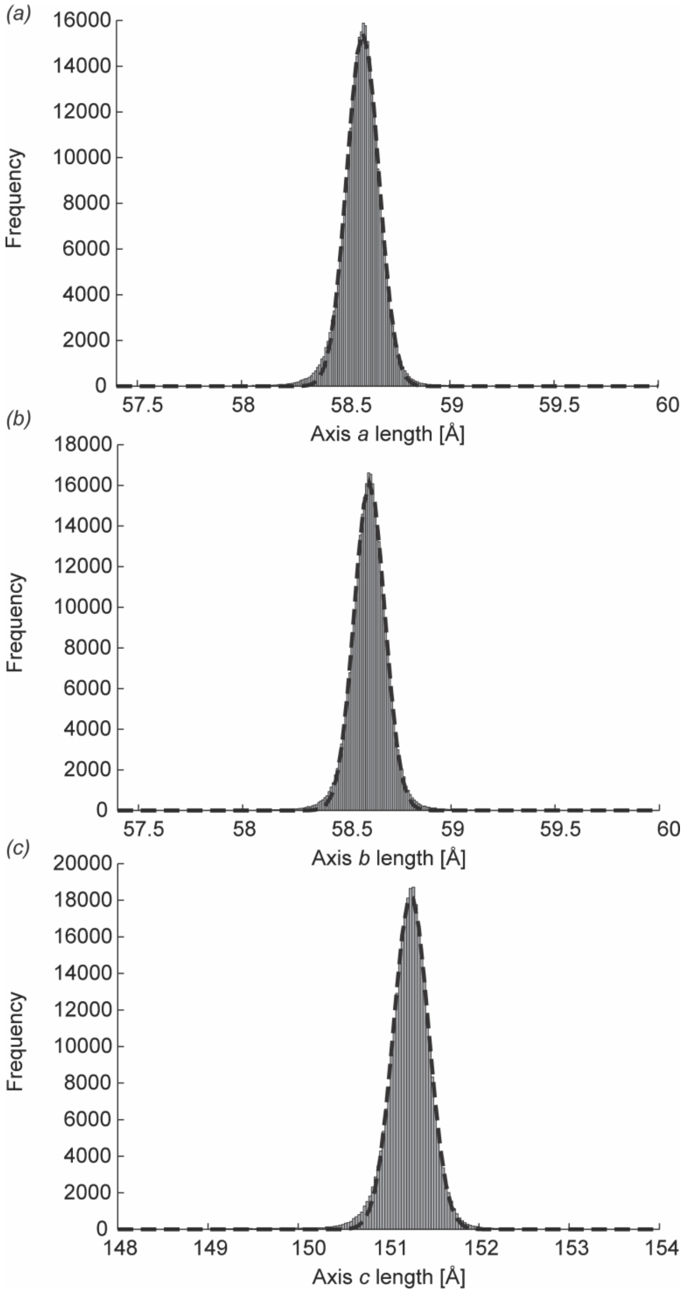

Figure S12 Comparison of data quality measures of thaumatin data sets after detector distance optimization with decreasing number of images. a) and b) show R_{split} and CC-1/2 values, c) and d) display CC* and CC_{ano} values, e) and f) show R_{ano}/R_{split} and signal-to-noise (SNR) values, g) displays the redundancy, i.e. the average number of measurements per resolution shell as a function of the resolution and number of indexed images used for Monte Carlo integration. It can be noticed that all data quality measures deteriorate with increasing resolution as well as with decreasing the number of indexed images used for integration.

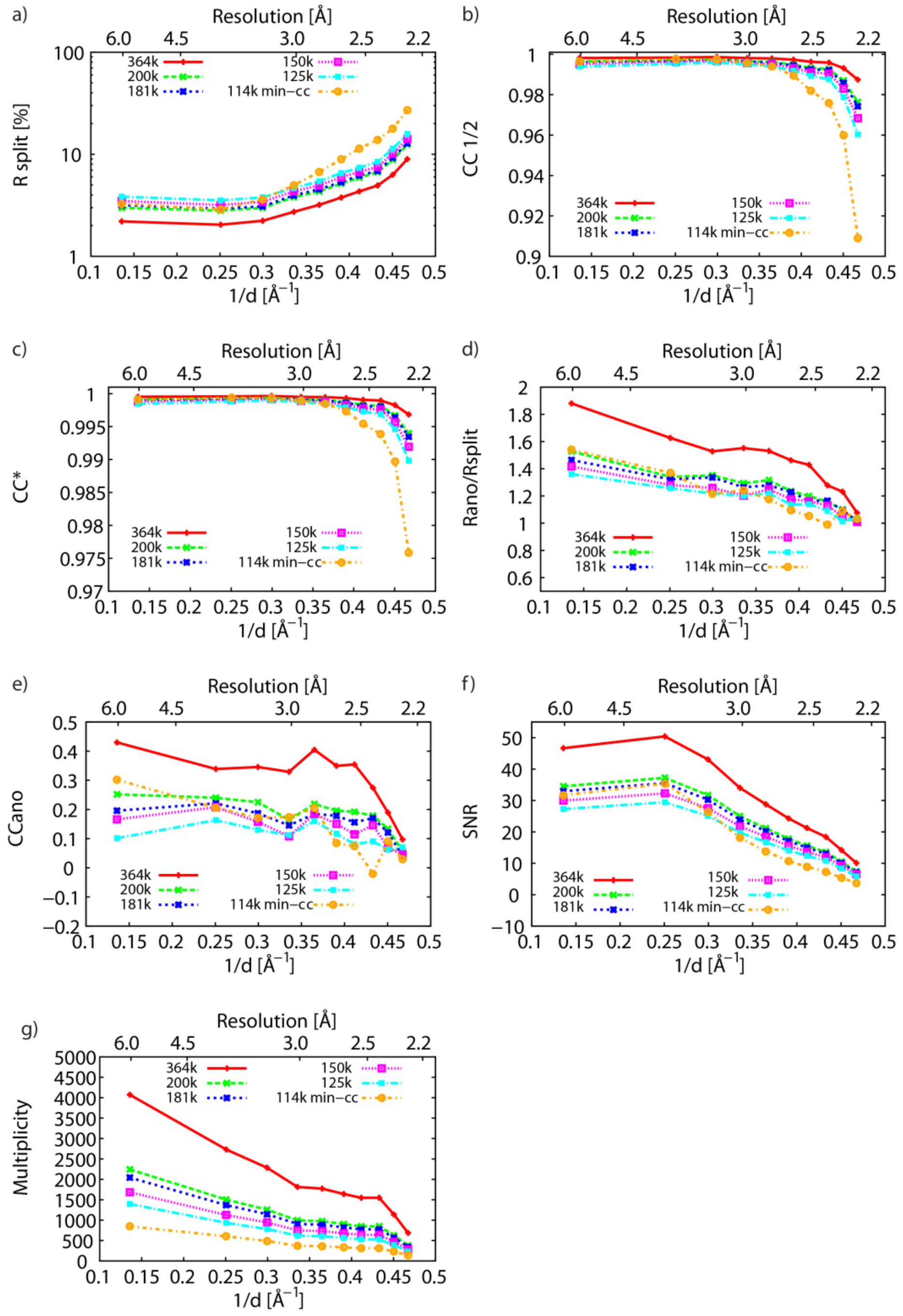

Figure S13 Plots with the overall data quality measures for thaumatin data sets with decreasing number of images in the resolution range 20 – 2.1 Å before (solid line, squares) and after (dashed line, circles) detector distance optimization and for the data set merged with CC_{min} =0.72 after detector distance optimization (triangle). a) and b)show R_{split} and CC-1/2 values, c) and d) CC* and CC_{ano} values, e) and f) R_{ano}/R_{split} and SNR values. The values for each data point are listed in Table S6. It is noticeable that most of the data quality measures improve after sample-to-detector distance optimization, in particular the signal to noise ratio, R_{split} and R_{ano}/R_{split} and SNR.

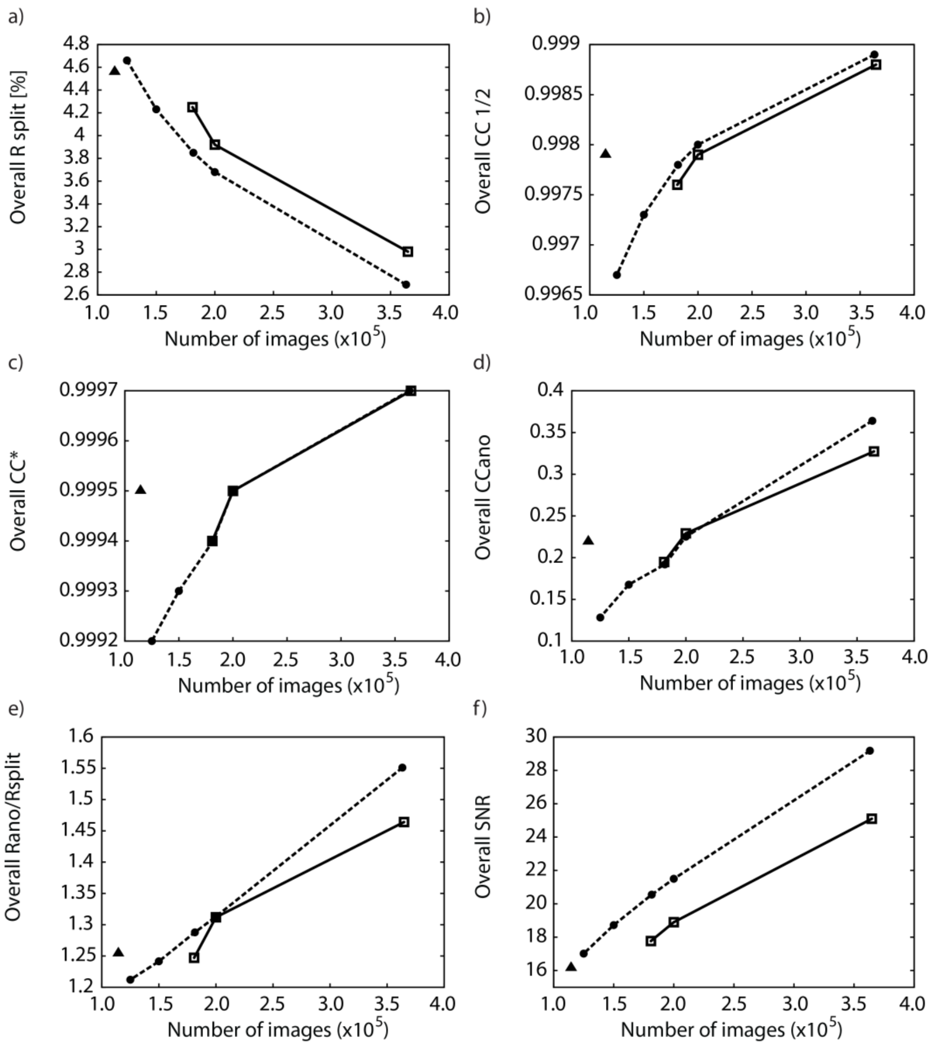

Figure S14 Plots of the anomalous signal-to-noise ratio a) “dano/sig” and b) “I/sig(I)” ratio from SHELXC as a function of resolution for thaumatin data sets containing different numbers of indexed images.

a)

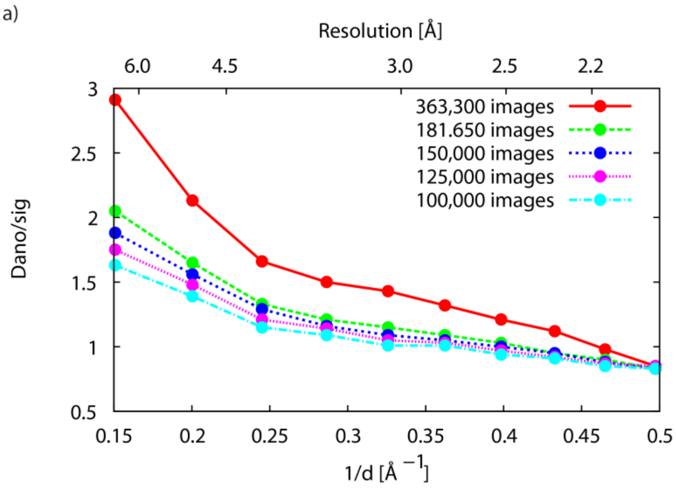

b)

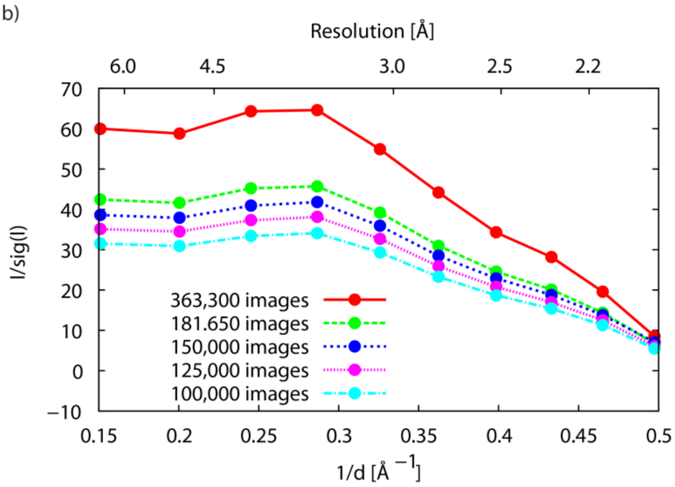

Figure S15 CC_{all} *vs* CC_{weak} plots from SHELXD for the thaumatin data sets after detector distance optimization. a) 10,000 trials and a resolution cut-off of 2.5 Å were used to find 17 S sites 16 of which could potentially be involved in disulphide bonds in the data set consisting of 363,300 images. Distinct clusters with correct substructure solutions were identified (red dots). The number of solutions with $CC_{all} \geq 30$ and $CC_{weak} \geq 9$ is 487. The density-modified map from SHELXE was used to trace almost the entire thaumatin structure using Autobuilt from Phenix (196 residues out of 207 were built with 195 sequenced correctly). This data set could also be phased and built in autoSHARP using a resolution limit of 2.2 Å. b) CC_{all} *vs* CC_{weak} plot from SHELXD for half the images (181,650) of the whole thaumatin data set after detector distance optimization. 10,000 trials were used to find 17 S sites of which 16 could potentially be involved in disulphide bonds; the resolution cut-off used for substructure determination was 3.5 Å. A distinct cluster with correct substructure solutions was identified. The number of solutions with $CC_{all} \geq 40$ and $CC_{weak} \geq 7$ is 119. c) CC_{all} *vs* CC_{weak} plot from SHELXD for 150,000 thaumatin images after detector distance optimization. 10,000 trials were used to find 17 S sites of which 16 could potentially be involved in disulphide bonds. The resolution cut-off for substructure determination was 3.5 Å. Distinct clusters with correct substructure solutions still can be identified. The number of solutions with $CC_{all} \geq 40$ and $CC_{weak} \geq 9$ is 108. This data set could be phased and built in autoSHARP using a resolution limit of 2.2 Å after filtering the top solutions from SHELXD with $CC_{all} \geq 45$ $CC_{weak} \geq 12$. d) CC_{all} *vs* CC_{weak} plot from SHELXD for 125,000 thaumatin images after detector distance optimization. 10,000 trials were used to find 17 S sites, 16 of which could potentially be involved in disulphide bonds. The resolution cut-off used for substructure determination was 3.5 Å. Distinct solutions with correct substructure solutions can be still identified. The number of solutions with $CC_{all} \geq 40$ and $CC_{weak} \geq 9$ is 10. This data set could be phased and built in autoSHARP using resolution limit of 2.2 Å after filtering the top solutions from SHELXD with $CC_{all} \geq 30$ $CC_{weak} \geq 12$. e) CC_{all} *vs* CC_{weak} plot from SHELXD for 100,000 thaumatin images after detector distance optimization. 500,000 trials were used to find 17 S sites of which 16 sites could potentially be involved in disulphide bonds. Although the maximum resolution used for substructure determination was 2.03 Å, no clear solutions with correct substructures were found.

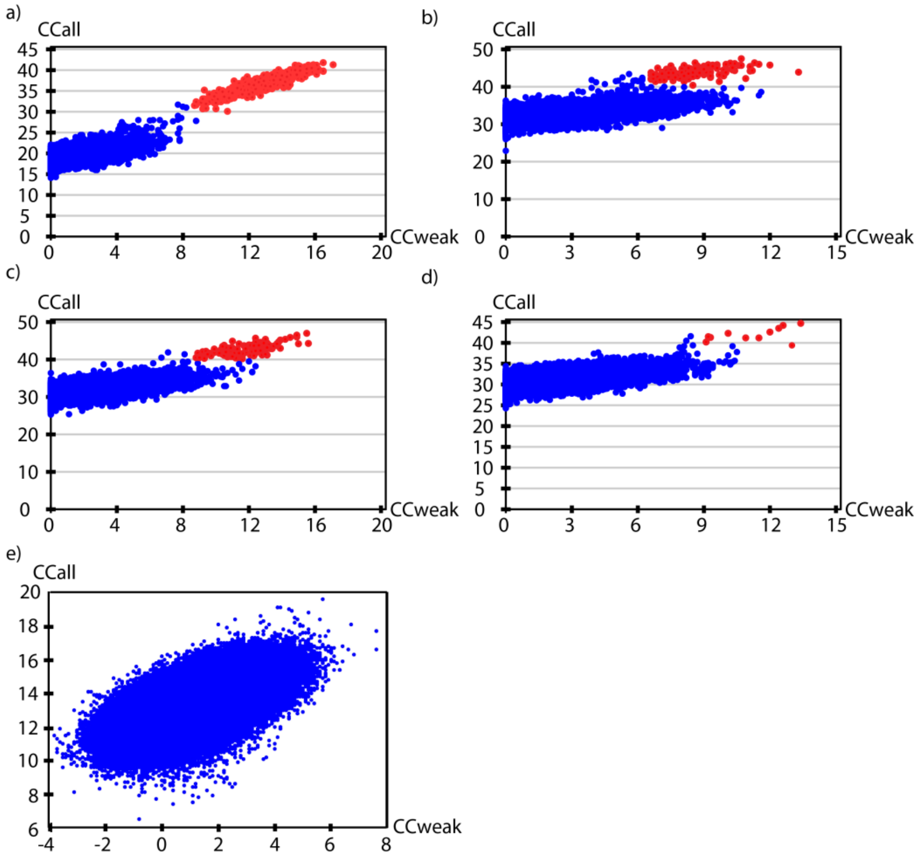
a)
CCall
CCweak
b)
CCall
CCweak
c)
CCall
CCweak
d)
CCall
CCweak
e)
CCall
CCweak

Figure S16 CC_{all} *vs* CC_{weak} plot from SHELXD for 114,540 images selected from the whole data set after detector distance optimization using a $CC_{min} \geq 0.72$ criterion. 10,000 trials were used to find 17 S sites, 16 of which could potentially be involved in disulphide bonds. The resolution cut-off used for substructure determination was 3.5 Å. A distinct cluster with correct substructure solutions (red dots) can be identified. The number of solutions with $CC_{all} \geq 35$ and $CC_{weak} \geq 10$ is 124. This data set could be phased and built in autoSHARP using resolution limit of 2.9 Å.

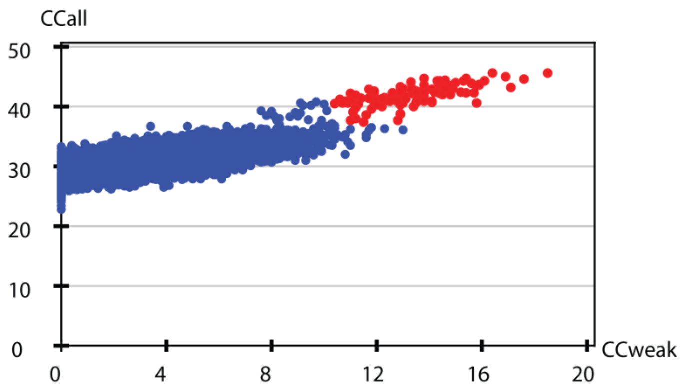

Figure S17 Experimental and simulated anomalous difference Patterson maps for various thaumatin data sets. The insets show w=1/2 sections of the anomalous difference Patterson maps from a) the reference thaumatin synchrotron dataset, b) all available SFX thaumatin images, c) 125,000 SFX images of thaumatin, d) calculated thaumatin data set. Data up to 2.0 Å resolution were included in the calculations. The maps are contoured at 1 sigma intervals.

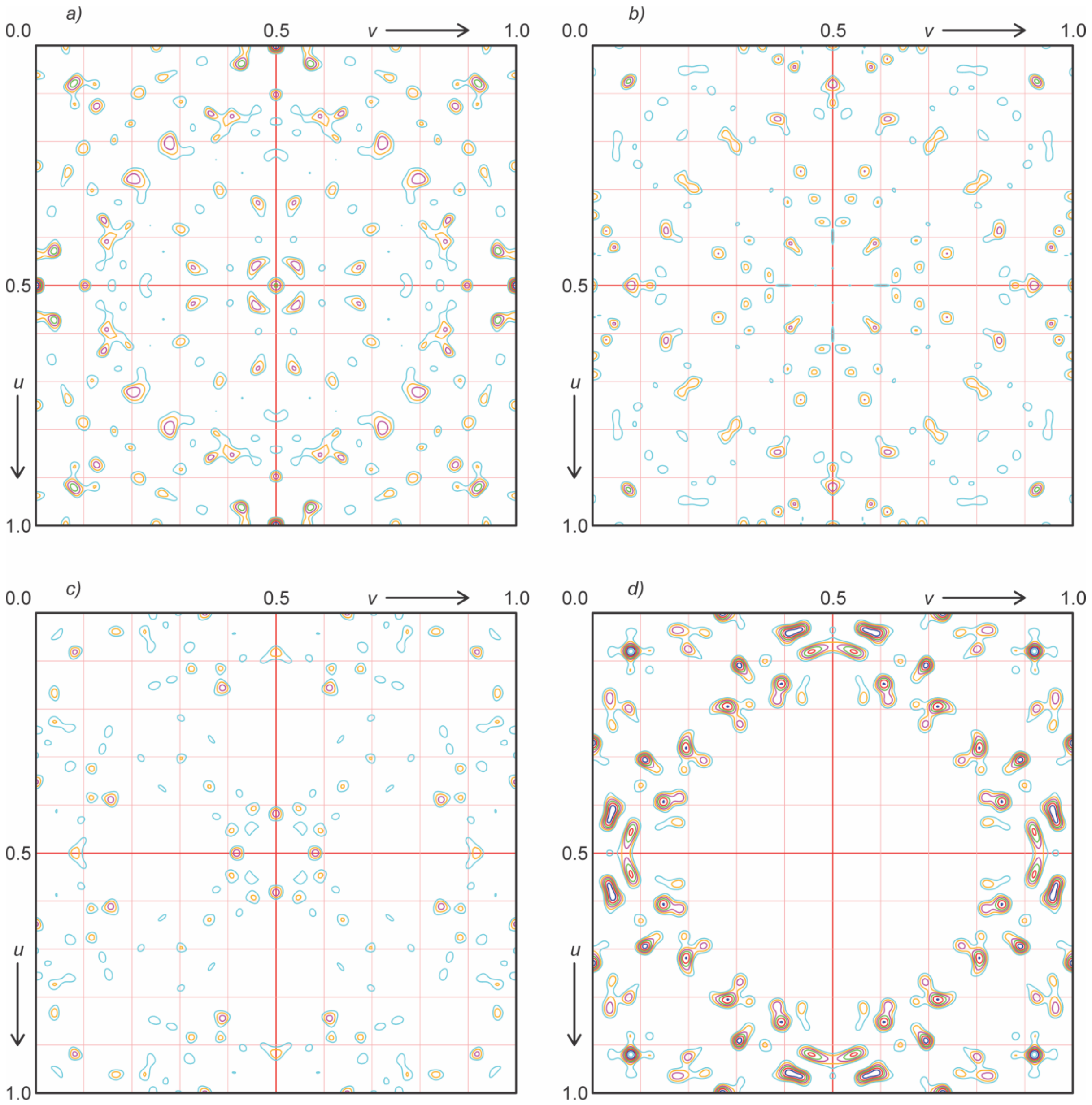

Table S1 Average unit cell parameters and corresponding detector distances of the lysozyme Gd-derivative data sets before and after detector distance optimization. The number of indexed images, unit cell lengths and the detector distance for the data set that contains all images indexed with the initial detector distance (112 mm) are given in the first row. The second row displays these data for the entire data set after detector distance optimization, and rows 3-5 for partial datasets from days (shifts) 1, 2, and 3, respectively. It should be noted that detector distance values obtained after refinement for different shifts may not be accurate since other factors such as wavelength or beam position changes were not taken into account.

Data set	# of images	*a* [Å]	*b* [Å]	*c* [Å]	det distance [mm]
All (previous, wrong det. dist.)	171,909	79.34 ± 0.5	79.05 ± 0.3	39.59 ± 0.3	112.00
All (days 1+2+3)	155,605	78.37 ± 0.2	78.27± 0.1	39.12± 0.1	Various
Day 1	47,858	78.32 ± 0.2	78.22 ± 0.1	39.12 ± 0.1	110.75
Day 2	48,983	78.37 ± 0.2	78.27 ± 0.1	39.12 ± 0.1	110.68
Day 3	58,764	78.32 ± 0.2	78.23 ± 0.1	39.06 ± 0.1	111.30

Table S2 Overall data quality statistics of the lysozyme Gd-derivative data sets before and after optimization of the detector distance for the resolution range 20 – 1.8 Å. Values given in parentheses refer to the highest resolution shell (1.9 – 1.8 Å). The first column gives the data set name, the second column lists the number of images included in the data set, and columns 3-8 give overall statistics for the whole resolution range. The second row and third rows refer to the data sets containing all indexed images before and after optimization of the detector distance, respectively. The optimization of the sample-to-detector distance was performed individually for three subsets of the whole data set collected on three different shifts ("day 1, day 2, day 3" in the bottom three rows). The improved quality of the whole data set after detector distance optimization is characterized by lower values of R_{split}, higher CC-1/2, CC*, R_{ano}/R_{split}, CC_{ano} and signal to noise ratio (SNR) than the whole data set before detector distance optimization, both for the whole resolution range and for the highest resolution shell, despite the reduced number of indexed images after detector distance optimization.

**Data set**	**Number of images**	**R_{split} [%]**	**CC ½**	**CC***	**R_{ano}/R_{split}**	**CC_{ano}**	**SNR**
All, before optimization	171,909	4.3 (64.9)	0.9977 (0.7379)	0.9994 (0.9215)	3.32 (1.10)	0.8189 (0.0848)	13.64 (1.50)
All, after optimization	155,605	4.2 (40.6)	0.9977 (0.8740)	0.9994 (0.9658)	3.43 (1.21)	0.8120 (0.2075)	14.94 (2.30)
Day 1	47,858	7.5 (65.1)	0.9925 (0.7278)	0.9981 (0.9178)	2.13 (1.05)	0.5633 (0.1018)	8.55 (1.47)
Day 2	48,983	7.4 (64.4)	0.9926 (0.7289)	0.9981 (0.9182)	2.12 (1.11)	0.5740 (0.1085)	8.64 (1.48)
Day 3	58,764	6.9 (86.3)	0.9939 (0.6215)	0.9985 (0.8755)	2.25 (1.10)	0.6148 (0.0874)	8.88 (1.14)

Table S3 Data quality measures for the lysozyme Gd-derivative data after detector distance optimization and as a function of a decreasing number of images All data subsets span the full resolution range (20 – 1.8 Å), with the number of images in the subset being randomly halved from one row to the next. The numbers given in parentheses refer to the highest resolution shell 1.9 – 1.8 Å.

**Number of images**	**R_{split} [%]**	**CC ½**	**CC***	**R_{ano}/R_{split}**	**CC_{ano}**	**SNR**
155,605	4.2 (40.6)	0.9977 (0.8740)	0.9994 (0.9658)	3.43 (1.21)	0.8120 (0.2075)	14.94 (2.30)
77,802	5.8 (52.0)	0.9956 (0.8077)	0.9989 (0.9453)	2.57 (1.08)	0.6776 (0.1113)	10.83 (1.83)
38,901	8.2 (71.9)	0.9909 (0.6883)	0.9977 (0.9030)	1.98 (1.05)	0.5179 (0.0978)	7.72 (1.33)
19,450	11.9 (104.2)	0.9800 (0.4815)	0.9949 (0.8062)	1.56 (1.05)	0.3489 (0.1057)	5.78 (0.97)
15,000	13.7 (116.8)	0.9744 (0.4153)	0.9935 (0.7661)	1.45 (1.07)	0.2868 (0.0698)	4.83 (0.87)
9,725	17.1 (154.3)	0.9605 (0.2216)	0.9899 (0.6024)	1.33 (1.04)	0.1929 (0.0143)	3.91 (0.72)
7,000	20.4 (187.1)	0.9444 (0.1840)	0.9856 (0.5574)	1.24 (1.05)	0.1167 (0.0356)	3.32 (0.62)
7,251 (CC_{min} ≥ 0.83)	16.6 (589.1)	0.9828 (0.0419)	0.9957 (0.2836)	1.37 (1.04)	0.3975 (-0.0241)	3.15 (0.17)

Table S4 Data quality measures for the lysozyme Gd-derivative data before detector distance optimization, as a function of a decreasing number of images. All data subsets span the full resolution range (20 – 1.8 Å), with the number of images in the subset being randomly halved from one row to the next.

**Number of images**	**R_{split} [%]**	**CC ½**	**CC***	**R_{ano}/R_{split}**	**CC_{ano}**	**SNR**
171,909	4.3	0.9977	0.9994	3.3168	0.8188	13.63
86,130	6.08	0.9953	0.9988	2.4709	0.6883	9.66
43,046	8.56	0.9912	0.9978	1.9008	0.5127	6.85
21,613	12.00	0.9828	0.9957	1.5462	0.3736	4.89
15,000	14.60	0.9741	0.9934	1.3790	0.2396	4.08
10,735	17.09	0.9658	0.9913	1.3140	0.2018	3.48
5,414	24.34	0.9310	0.9820	1.1573	0.0650	2.53

Table S5 Phase improvement by SHELXE for lysozyme Gd data sets using different number of indexed images before detector optimization at 1.8 Å resolution assuming 0.43 solvent content after 80 cycles.

Number of indexed images	enantiomer	contrast[a]	Pseudo-free CC[b]	Number of residues[c]	$CC_{(trace)}$[d]
171,909	original hand<br>*inverted hand*	0.44<br>*0.34*	72.9<br>*50.3*	122<br>*35*	51.7<br>*11.3*
86,130	original hand<br>*inverted hand*	0.45<br>*0.35*	72.1<br>*49.5*	122<br>*23*	49.21<br>*11.9*
43,046	original hand<br>*inverted hand*	0.45<br>*0.37*	70.0<br>*50.4*	114<br>*24*	46.2<br>*11.6*
21,613	original hand<br>*inverted hand*	0.47<br>*0.40*	62.1<br>*40.5*	108<br>*15*	43.4<br>*11.2*
10,735	original hand<br>*inverted hand*	0.39<br>*0.32*	63.0<br>*n.d.*	102<br>*n.d.*	41.5<br>*n.d*
5,414	original hand<br>*inverted hand*	0.45<br>*0.38*	49.6<br>*47.3*	29<br>*17*	9.6<br>*7.4*

[a] solvent to protein region contrast as defined by SHELXE (Sheldrick, 2002)

[b] correlation coefficient between 10 % randomly selected E_{obs} and their E_{calc} after one cycle of density modification in which the selected normalized structure factor amplitudes were omitted.

[c] number of residues of a poly-alanine model assigned to the electron density map after 3 cycles of auto-tracing by SHELXE.

[d] correlation coefficient for the partial structure against data.

Table S6 Phase improvement by SHELXE for lysozyme Gd data sets using different number of indexed images after detector optimization at 1.8 Å resolution assuming 0.43 solvent content after 80 cycles.

Number of indexed images	enantiomer	contrast[a]	Pseudo-free CC[b]	Number of residues[c]	$CC_{(trace)}$[d]
155,605	original hand<br>*inverted hand*	0.43<br>*0.36*	72.6<br>*48.4*	120<br>*33*	49.7<br>*10.7*
77,802	original hand<br>*inverted hand*	0.45<br>*0.38*	73.9<br>*55.7*	121<br>*23*	47.3<br>*10.8*
38,901	original hand<br>*inverted hand*	0.47<br>*0.37*	68.3<br>*46.9*	108<br>*28*	46.4<br>*11.1*
19,450	original hand<br>*inverted hand*	0.47<br>*0.39*	67.1<br>*50.0*	115<br>*25*	47.9<br>*11.5*
15,000	original hand<br>*inverted hand*	0.47<br>*0.39*	65.9<br>*49.2*	117<br>*19*	48.3<br>*8.13*
9,725	original hand<br>*inverted hand*	0.49<br>*0.43*	65.8<br>*53.6*	109<br>*23*	44.1<br>*11.8*
7,000	original hand<br>*inverted hand*	0.51<br>*0.45*	63.1<br>*49.3*	93<br>*15*	36.1<br>*11.0*
7,252<br>$CC_{min} \geq 0.83$	original hand<br>*inverted hand*	0.43<br>*0.35*	58.7<br>*48.45*	96<br>*31*	35.3<br>*7.63*

[a] solvent to protein region contrast as defined by SHELXE (Sheldrick, 2002)*

[b] correlation coefficient between 10 % randomly selected E_{obs} and their E_{calc} after one cycle of density modification in which the selected normalized structure factor amplitudes were omitted.

[c] number of residues of a poly-alanine model assigned to the electron density map after 3 cycles of auto-tracing by SHELXE.

[d] correlation coefficient for the partial structure against data.

* Sheldrick, G.M. (2002). *Z. Kristallogr*. **217**, 644-650.

Macromolecular phasing with SHELXE

Table S7 Data quality measures for thaumatin data sets containing different numbers of indexed images for the whole resolution range 20 – 2.1 Å before and after detector distance optimization. The values given in parentheses refer to the highest resolution shell 2.0 – 2.1 Å.

**Number of images**	**R_{split} [%]**	**CC ½**	**CC***	**R_{ano}/R_{split}**	**CC_{ano}**	**SNR**
364,782 (before)	2.98 (17.66)	0.9988 (0.9633)	0.9997 (0.9906)	1.4640 (1.0082)	0.3273 (-0.0443)	25.10 (5.09)
200,000 (before)	3.92 (23.28)	0.9979 (0.9372)	0.9995 (0.9837)	1.3119 (1.0248)	0.2291 (-0.0189)	18.90 (3.89)
180,964 (before)	4.25 (25.19)	0.9976 (0.9287)	0.9994 (0.9813)	1.2472 (0.9855)	0.1948 (-0.0288)	17.77 (3.61)
363,300 (after)	2.69 (8.96)	0.9989 (0.9874)	0.9997 (0.9968)	1.5509 (1.0765)	0.3640 (0.0973)	29.17 (10.11)
200,000 (after)	3.68 (12.48)	0.9980 (0.9764)	0.9995 (0.9940)	1.3130 (1.0076)	0.2251 (0.0565)	21.50 (7.42)
181,650 (after)	3.85 (12.80)	0.9978 (0.9743)	0.9994 (0.9935)	1.2879 (1.0149)	0.1919 (0.0406)	20.55 (7.15)
150,000 (after)	4.23 (14.17)	0.9973 (0.9684)	0.9993 (0.9919)	1.2414 (1.0090)	0.1678 (0.0462)	18.73 (6.55)
125,000 (after)	4.66 (15.85)	0.9967 (0.9604)	0.9992 (0.9898)	1.2122 (1.0395)	0.1284 (0.0705)	17.02 (5.87)
114,540 (after, $CC_{min} \geq 0.72$)	4.56 (27.07)	0.9979 (0.9089)	0.9995 (0.9758)	1.2541 (1.0310)	0.2192 (0.0295)	16.16 (3.71)

Table S8 Phase improvement by SHELXE for thaumatin data sets using different numbers of indexed images after detector optimization at 2.0 Å resolution assuming 0.56 solvent content after 80 cycles.

Number of indexed images	enantiomer	contrast[a]	Pseudo-free CC[b]	Number of residues[c]	$CC_{(trace)}$[d]
363,300	original hand<br>*inverted hand*	0.71<br>*0.31*	70.6<br>*41.0*	176<br>*85*	43.5<br>*12.5*
181,650	original hand<br>*inverted hand*	0.49<br>*0.31*	44.9<br>*38.4*	97<br>*67*	15.9<br>*10.2*
150,00	original hand<br>*inverted hand*	0.43<br>*0.41*	47.4<br>*43.6*	69<br>*41*	14.0<br>*9.3*
125,000	original hand<br>*inverted hand*	n.d<br>n.d	n.d<br>n.d	n.d<br>n.d	n.d<br>n.d
114,540<br>(CC_{min}≥0.72)	original hand<br>*inverted hand*	0.36<br>*0.34*	41.4<br>*40.9*	62<br>*84*	11.2<br>*12.9*

[a] solvent to protein region contrast as defined by SHELXE (Sheldrick, 2002)

[b] correlation coefficient between 10 % randomly selected E_{obs} and their E_{calc} after one cycle of density modification in which the selected normalized structure factor amplitudes were omitted.

[c] number of residues of a poly-alanine model assigned to the electron density map after 3 cycles of auto-tracing by SHELXE.

[d] correlation coefficient for the partial structure against data.
